# Yiqihuoxue decoction (GSC) inhibits mitochondrial fission through the AMPK pathway to ameliorate EPCs senescence and optimize vascular aging transplantation regimens

**DOI:** 10.1186/s13020-024-01008-7

**Published:** 2024-10-14

**Authors:** Yinan Liu, Zenghui Niu, Xue Wang, Chengkui Xiu, Yanhong Hu, Jiali Wang, Yan Lei, Jing Yang

**Affiliations:** 1 Beijing Key Laboratory of Traditional Chinese Medicine Basic Research on Prevention and Treatment for Major Diseases, Experimental Research Center, China Academy of Chinese Medical Sciences, Beijing, 100700 China; 2 Graduate School of China Academy of Chinese Medical Sciences, Beijing, 100700 China; 3 Tianjin Academy of Traditional Chinese Medicine Affiliated Hospital, Tianjin, 300120 China

**Keywords:** Vascular aging, GSC, Mitochondrial fission, Transplantation, AMPK

## Abstract

**Background:**

During the aging process, the number and functional activity of endothelial progenitor cells (EPCs) are impaired, leading to the unsatisfactory efficacy of transplantation. Previous studies demonstrated that Yiqihuoxue decoction (Ginseng-Sanqi-Chuanxiong, GSC) exerts anti-vascular aging effects. The purpose of this study is to evaluated the effects of GSC on D-galactose (D-gal)induced senescence and the underlying mechanisms.

**Methods:**

The levels of cellular senescence-related markers P16, P21, P53, AMPK and p-AMPK were detected by Western blot analysis (WB). SA-β-gal staining was used to evaluate cell senescence. EPCs function was measured by CCK-8, Transwell cell migration and cell adhesion assay. The morphological changes of mitochondria were detected by confocal microscopy. The protein and mRNA expression of mitochondrial fusion fission Drp1, Mff, Fis1, Mfn1, Mfn2 and Opa1 in mitochondria were detect using WB and RT–qPCR. Mitochondrial membrane potential, mtROS and ATP of EPCs were measured using IF. H&E staining was used to observe the pathological changes and IMT of the aorta. The expressions of AGEs, MMP-2 and VEGF in aorta were measured using Immunohistochemical (IHC). The levels of SOD, MDA, NO and ET-1 in serum were detected by SOD, MDA and NO kits.

**Results:**

In vitro, GSC ameliorated the senescence of EPCs induced by D-gal and reduced the expression of P16, P21 and P53. The mitochondrial morphology of EPCs was restored, the expression of mitochondrial Drp1, Mff and Fis1 protein was decreased, the levels of mtROS and ATP were decreased, and mitochondrial function was improved. Meanwhile, the expression of AMPK and p-AMPK increased. The improvement effects of GSC on aging and mitochondrial morphology and function were were hindered after adding AMPK inhibitor. In vivo, GSC improved EPCs efficiency, ameliorated aortic structural disorder and decreased IMT in aging mice. The serum SOD level increased and MDA level decreased, indicating the improvement of antioxidant capacity. Increased NO content and ET-1 content suggested improvement of vascular endothelial function. The changes observed in SOD and MMP-2 suggested a reduction in vascular stiffness and the degree of vascular damage. The decreased expression of P21 and P53 indicates the delay of vascular senescence.

**Supplementary Information:**

The online version contains supplementary material available at 10.1186/s13020-024-01008-7.

## Introduction

Cardiovascular disease (CVD) is an important factor affecting human health [1], and age is an important indicator of cardiovascular health [2]. Vascular aging precedes aging, but aging can accelerate the process of vascular aging. The structure and function of arteries are altered with age [3]. The inner arterial membrane, which is composed of endothelial cells (ECs), is the first barrier that protects the blood vessel wall, while endothelial progenitor cells (EPCs) are bone marrow stem cells and precursor cells of ECs that can be used as treatments for diseases associated with endothelial integrity and dysfunction. Although autologous EPCs transplantation and mobilization of EPCs by drugs contribute to vascular repair and neovascularization [4], the number and functional activity of EPCs are impaired by aging, and the efficacy of transplantation is not satisfactory. Therefore, there is an urgent need to identify an effective drug and develop a better method for autologous EPCs transplantation.

Mitochondrial dysfunction has an important impact on the aging of organisms and is a preliminary aging marker [5]. Activation of AMPK inhibits mitochondrial ectasia in Drpl and reduces mitochondrial fragmentation and reactive oxygen species (ROS) production, which in turn ameliorates senescence-induced increases in inflammatory factor expression and apoptosis [7]. Notably, there is also a decrease in AMPK levels and activity during senescence. An important strategy for ameliorating cellular senescence and related diseases is to regulate the dynamic balance of mitochondrial fission and fusion via the AMPK pathway.

GSC is a traditional Chinese medicine formula based on the experience of Academician Keji Chen of Xiyuan Hospital in treating coronary heart disease [9–11], including Ginseng (*Panax ginseng* C.A.Mey.), San Qi (*Panax notoginseng* (Burk.) F.H.Chen.) and Chuan Xiong (*Ligusticum sinense 'Chuanxiong' Hort.*). The signature chemical components of GSC are ginsenosides Rg_1_, Rb_1_, and Re; the Panax ginseng saponin R_1_; and ferulic acid. Our previous studies have shown that GSC exerts anti-vascular aging effects in animal and ECs models through multiple pathways, such as the mitochondrial autophagy and intestinal flora [14–16]. More importantly, our recent study demonstrated that GSC enhance the function and delay senescence of autologous EPCs [17], but the mechanism underlying the delay of EPCs senescence remains unclear. Based on our previous studies, we hypothesize that GSC mitigates the senescence of EPCs by regulating the AMPK pathway, that autografting EPCs improves aortic senescence in mice to affect mitochondrial fusion/fission and that GSC enhances the therapeutic effect of transplanted EPCs.

**Fig. 1:**
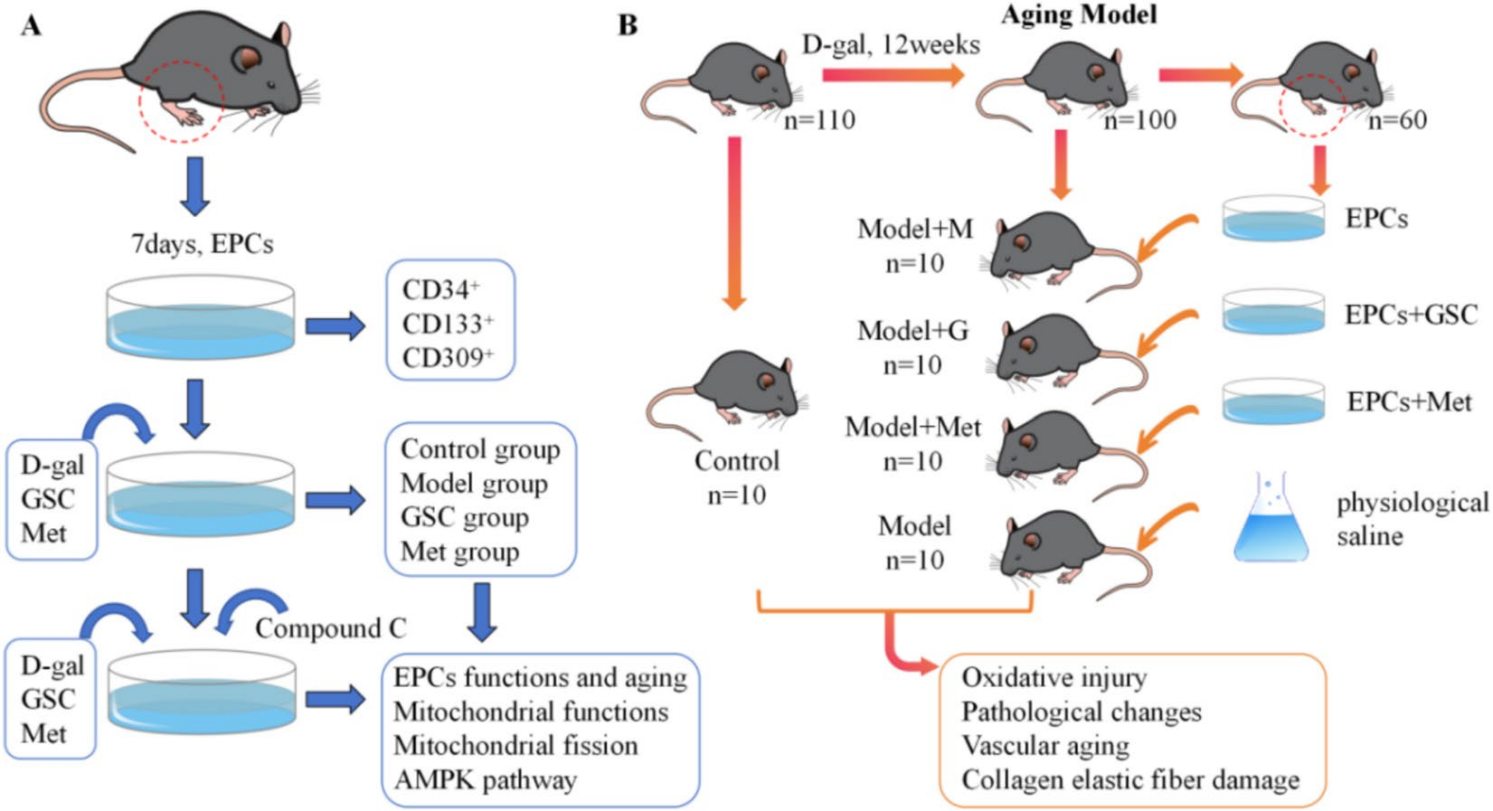
The design of the experiment.** A** In vitro experiment. EPCs were treated in medium containing D-gal and different concentrations of GSC and Met for 24h. Then the EPCs were divided into Control, Model, GSC and Met groups. The mitochondrial function, morphology and fusion division related gene alterations and protein expression were detected in each group. We examined the expression of AMPK pathway proteins. The groups were pre-treated with Compound C 5µm for 1h. **B** Autotransplantation experiments. We built the model of aging mice by dorsal cervical injections of D-gal. 60 aging mice were randomly selected and their bone marrow cells were extracted and induction cultured. In vitro intervention of EPCs using GSC, Met, and EGM-2MV culture medium. 40 model mice were assigned to Model, Model + M, Model + GSC, and Model + Met groups (n = 10 per group). 14 days after administration of EPCs, mice were sacrificed

The schematic design of this study is shown in Fig. 1A. Briefly, we treated EPCs with different concentrations of D-gal based on previous methods [18]. The EPCs were subsequently divided into four groups. Mitochondrial function, morphology and fusion/fission-related gene alterations and protein expression were determined in each group. To investigate the underlying mechanisms, we examined the expression of AMPK pathway-related proteins. The groups were pretreated with Compound C to observe the effects of inhibiting the AMPK pathway on the function, senescence, mitochondrial function, morphology and mitochondrial fusion and fission-related proteins of EPCs.

Furthermore, we explored the effect of GSC in an in vitro intervention using EPCs on improving vascular senescence via autografts in senescent mice. In accordance with previous studies [19, 20], we generated a model of aging mice by dorsal cervical injection of D-gal. After modeling, EPCs from senescent mice were cultured in vitro in GSC, Met, or culture medium. The EPCs in each group were then transplanted via tail vein injection. Finally, we assessed vascular senescence in mice by detecting changes in oxidative stress, endothelial function, aortic morphology, aortic atherosclerosis, and senescence-related protein expression.

## Materials and methods

### Animals and drug

Six-week-old male C57BL/6N mice were obtained from Beijing Vital River Laboratory Animal Technology Co., Ltd. (Beijing, China, SYXK2017-0033) and maintained under specific pathogen-free conditions in the Animal Experimental Center of the Institute of Basic Theory for Chinese Medicine of the China Academy of Chinese Medical Sciences. The mice were maintained at a temperature of 22 ± 2 °C and a humidity of 60 ± 5% on a regular 12-h light/dark cycle. All animal experimental protocols and procedures in this study were approved and performed in accordance with the guidelines of the Committee of the Animal Welfare Ethics of the Institute of Basic Theory for Chinese Medicine of the China Academy of Chinese Medical Sciences of Health Guidelines on the Care and Use of Animals (Approval No. 2020–11).

GSC is composed of Ginseng (*Panax ginseng C.A.Mey.*), San Qi (*Panax notoginseng (Burk.) F.H.Chen.*) and Chuan Xiong (*Ligusticum sinense 'Chuanxiong' Hort.*). Detailed assays and preparation methods are described in **Supplementary Materials and methods 1.1**. The content of the hallmark ingredients in GSC was determined using HPLC (**Supplementary Fig. 1–5, Table 1–5**). Metformin (Met) hydrochloride (serial number: D9351) was obtained from Solarbio & Science Technology (Beijing) Co., Ltd. D-galactose (serial number: G0750-100G) was purchased from US Sigma‒Aldrich.

### Aging animal model preparation and EPCs transplantation

As reported previously [19, 20], the aging model was established in mice by dorsal cervical injections of D-gal (180 mg/kg/day) for 11 weeks. Autotransplantation of EPCs was performed as previously described [21, 22], 60 aging mice were randomly selected and their bone marrow cells were extracted. After 7 days of induction culture, EPCs were divided equally into three groups, which were subjected to 24h intervention using EGM-2MV culture medium, GSC and Met, respectively. After that the EPCs were digested and resuspended using saline. The remaining 40 model mice were assigned to the Model with physiological saline (Model), Model with EPCs (Model + M), Model with EPCs + GSC (Model + GSC), and Model with EPCs + Met (Model + Met) groups. EPCs were transfected using lentiviral vectors with double reporter genes of luciferase and enhanced green fluorescent protein (HBLV-ZsGreen-LUC-PURO, Hanbio Biotechnology Co., Ltd., Shanghai, China). One day after administration of EPCs (1 × 106 cells, tail vein), the mice were observed using In Vivo Imaging System (PERKINELMER, USA, IVIS Lumina III). Mice were sacrificed to collect serum and aortic in 14 days. (Fig. 1** B**).

### Mitochondrial-specific fluorescence staining

EPCs were seeded in glass bottom cell culture dishes (2000 cells/well) and treated with MitoTracker Red CMRos (Invitrogen, USA, M7512) according to the manufacturer’s protocol. Observation was performed using a confocal microscope (Olympus, Japan, FV1000) at a wavelength of 579/599 nm for excitation and emission light maxima.

### Real-time reverse transcription-quantitative polymerase chain reaction (RT–qPCR)

Total RNA was extracted from the EPCs using a TransZol UP Kit (TransGen, Beijing, ET111) according to the manufacturer’s instructions [27]. Then, the corresponding cDNA was synthesized using a TransScript First-Step RT‒PCR SuperMix Kit (TransGen, Beijing, AT311). Quantitative analysis of the messenger RNAs (mRNAs) of Drp1, Mff, Fis1, Opa1, Mfn1, and Mfn2 was performed using a real-time PCR instrument (Bio-Rad, USA, T100) with SYBR® Green Real-time PCR Master Mix (Toyobo, Japan, QPK-201T). The reaction conditions consisted of predenaturing at 95 °C for 30 s, followed by 40 cycles of 95 °C for 5 s, 55 °C for 30 s and 72 °C for 15 s. The relative expression of the target genes was compared with that of β-actin, an endogenous reference gene, and calculated using the 2^−ΔΔCt^ method. The primers used were obtained from Bioengineering Shanghai Co., Ltd. The sequences of primers used for RT‒qPCR is shown in Table 1.

**Table 1:** Primer sequences for qPCR

Gene	Forward	Reverse
*Drp1*	GGG CGA ACC TTA GAA TCT GTG GAC	ATG GCA TCG TGA AGT TTA GGG AAC C
*Mff*	CAC CAC CAA ATG CTG ACC TGG AG	TTC GCT TTG AGG AGT TGG AAG TGG
*Fis1*	GCA AAG AGG AAC AGC GGG ACT ATG	TCA GGA TTT GGA CTT GGA GAC AGC
*Opa1*	CGT TCA CAT CAT CCT GCC CTC AGG	TAG CCA CCT CCA ACA GCA GAT CC
*Mfn1*	GAG TGT ATC TCG CAG TCA GCA GTG	TCC TCC GTG ACC TCC TTG ATC TTC
*Mfn2*	GCA TTC TTG TGG TCG GAG GAG TG	TTT GGC TCT GCT CTG AAG TGA ATC C
*β-actin*	TGC TGT CCC TGT ATG CCT CTG G	ACC GCT CGT TGC CAA TAG TGA TG

### Western blot analysis

The proteins in the lysed samples (Solarbio, China, R0010) were quantified with a BCA protein quantification kit (Solarbio, China, PC0020) according to the manufacturer's instructions. Afterward, SDS‒PAGE upsampling buffer was added to each sample, and the samples were boiled for 5 min. Equal amounts of protein (30 μg) were separated on a 12% SDS‒PAGE gel and then transferred to a PVDF membrane by electrophoresis (Millipore, USA; IPVH00010). The membrane was then blocked with 5% skim milk or 5% BSA for 1h at room temperature and incubated overnight at 4 °C with the corresponding primary antibodies against P53 (1:1000; Proteintech, China; 60,282–2-lg), P21 (1:1000; Proteintech, China; 28,248–1-AP), P16 (1:10,000; Abcam, USA; Ab211542), Drpl (1:2000; Proteintech, China; 12,957–1-AP), Mff (1:2000; Proteintech, China; 17,090–1-AP), Fis1 (1:1000; Proteintech, China; 10,956–1-AP), Opal (1:2000; Proteintech, China; 27,733–1-AP); Mfn1 (1:2000; Proteintech, China; 13,798–1-AP); Mfn2 (1:2000; Proteintech, China; 12,186–1-AP); AMPK (1:2500; Abcam, USA; Ab32047); phosphorylated (p)-AMPK (1:2000; CST, USA; 2535T); or β-actin (1:20,000; Proteintech, China; 81,115–1-RR); and GAPDH (1:20,000; Proteintech, China; 60,004–1-lg). After three washes with TBST, the membranes were incubated with anti-rabbit or anti-mouse secondary antibodies (1:500; SeraCare, USA; 5220–0341, 5220–0336) for 1 h at room temperature. An enhanced chemiluminescence detection system was used to visualize the immunoreactive bands.

### Hematoxylin and eosin (H&E) staining

Mouse aortic tissues were fixed in 4% neutral formalin solution, embedded in paraffin and cut into 4 µm thick sections. Finally, H&E staining was performed, and histopathological changes in the aorta were observed under a light microscope. The intima-media thickness (IMT) of the aorta was measured using OLYMPUS OlyVIA 2.9 software; at least 5 fields of view were examined per mouse.

### Immunohistochemical (IHC) analyses

IHC staining was performed as previously reported [28]. Briefly, aortic sections were prepared, rehydrated and subjected to antigen recovery with sodium citrate. After blocking with 3% BSA for 30 min, the sections were incubated in a humidified chamber at 4 °C with specific antibodies against P53 (1:3200; Proteintech Group, USA; 60,283-2Ig), AGEs (1:100; Proteintech Group, USA; 19,003–1-AP), MMP-2 (1:400; Proteintech Group, USA; 10,373–2-AP). The specimens were then treated with secondary antibiotics and diaminobenzidine (Jiangsu Shitai Experimental Equipment Co., Ltd., Jiangsu, China, 2,005,289). Five fields of view were selected for imaging of each section under an Olympus BX61VS microscope at a magnification of × 200.

### Statistical analysis

All the data are presented as the means ± SDs. Statistical analysis was conducted via one-way ANOVA using SPSS 20.0 software (IBM, New York, NY, USA). The calculation of the area of positive expression by IHC staining and Western blotting was conducted using ImageJ software (NIH, USA). Differences were considered significant at *P* < 0.05, *P* < 0.01, and *P* < 0.001.

## Results

### GSC delays EPCs senescence by improving mitochondrial function and inhibiting excessive fission

Studies have suggested that changes in mitochondrial dynamics are one of the important features of animal and cellular aging [5]. We not only isolated and characterized EPCs derived from mice bone marrow stem cells (**Supplementary Fig. 6**), but also assessed the impact of D-gal, Met and GSC on cellular viability and senescence (**Supplementary Fig. 7–9**). Our research has been demonstrated that both GSC and Met possess the capability to mitigate the aging of EPCs induced by D-gal (**Supplementary Fig. 10**).

**Fig. 2:**
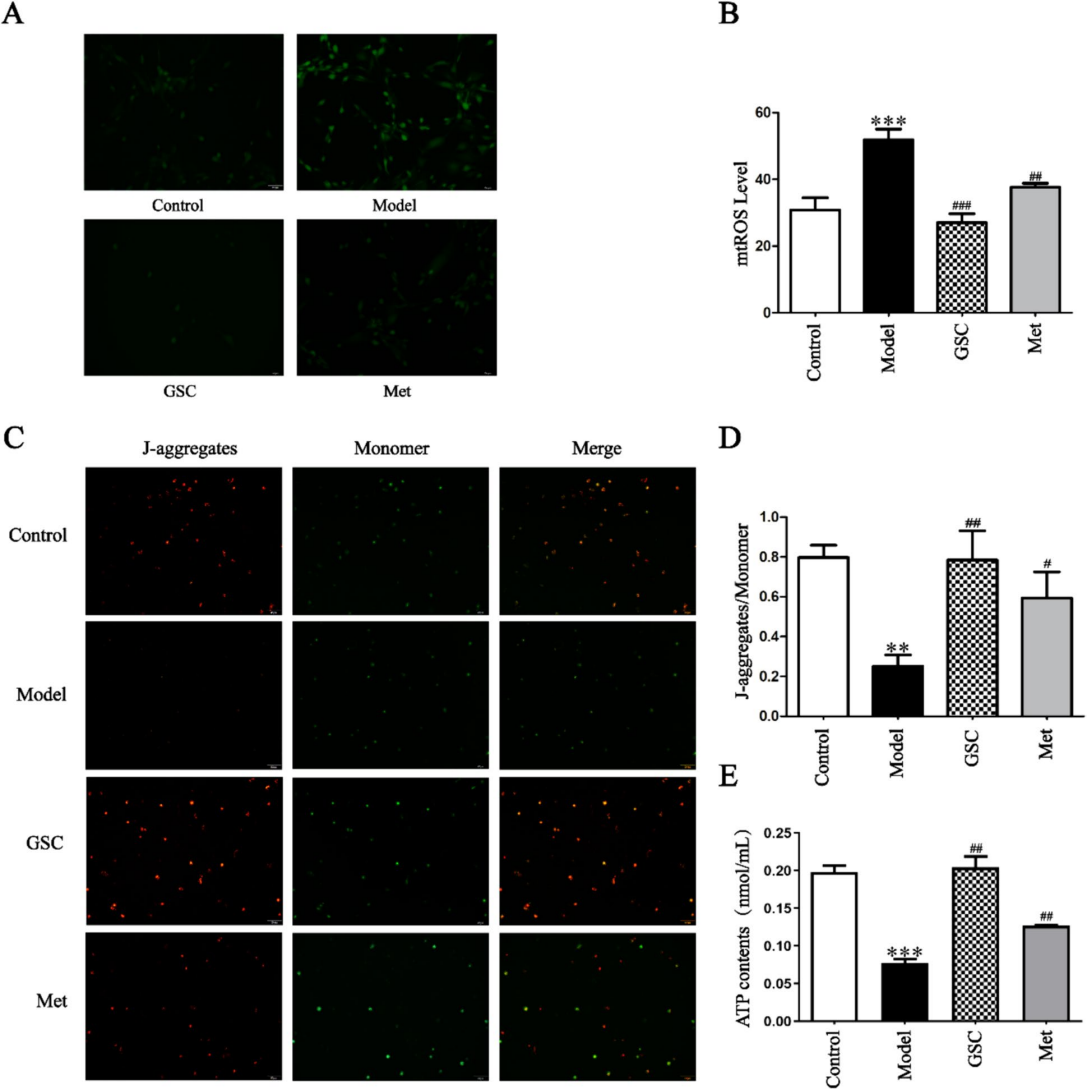
Effects of GSC on mitochondrial function in EPCs.** A**, **B** Effect of GSC on ROS levels in EPCs (200x). **C**-**D** Effects of GSC on the MMP in EPCs (200x). **E** Effect of GSC on the ATP content in EPCs. The scale is 50 μm. Compared with those in the control group, **P < 0.01, ***P < 0.001; Compared with those in the model group, ^#^*P* < 0.05, ^##^*P* < 0.01, ^###^*P* < 0.001

**Fig. 3:**
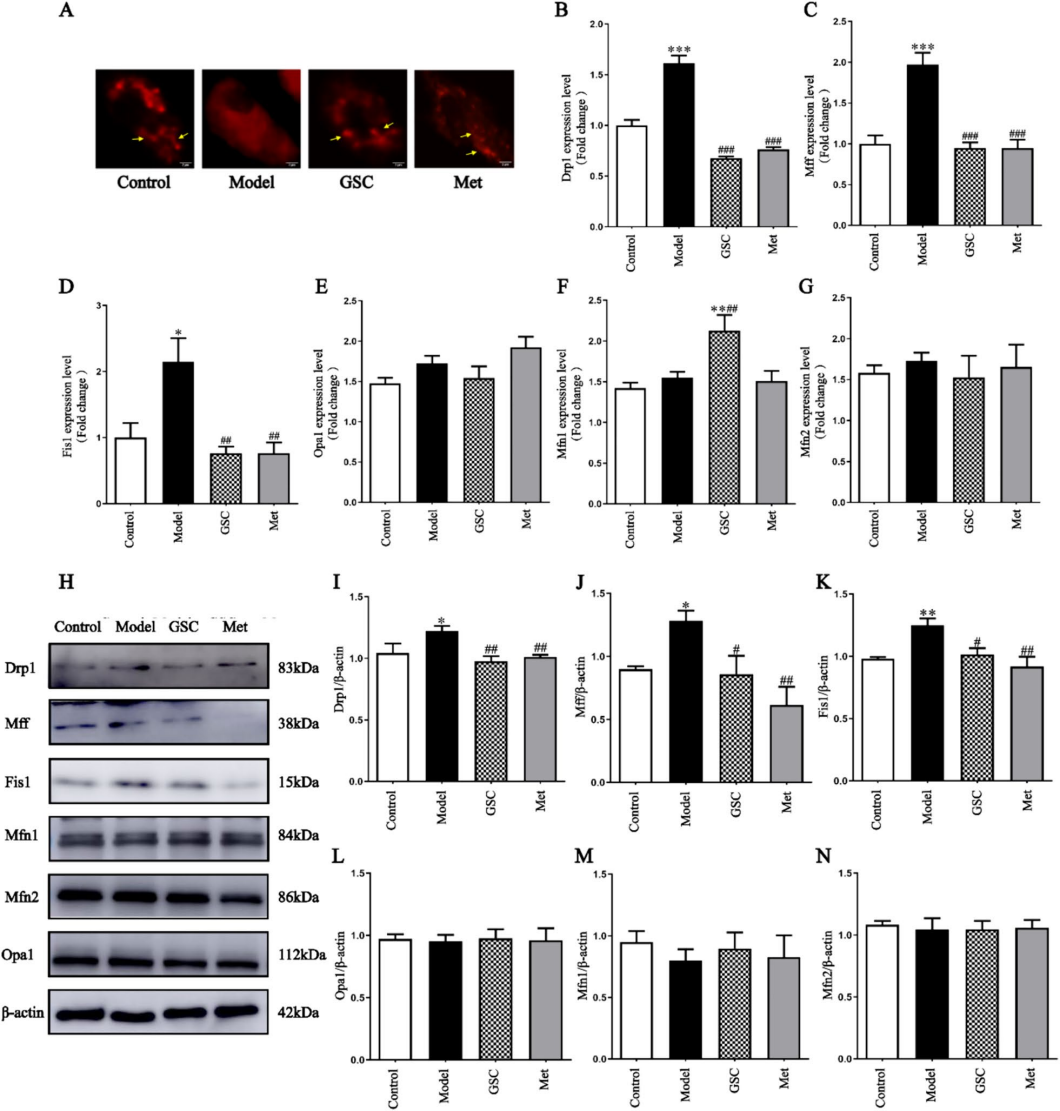
Effect of GSC on mitochondrial fusion and fission in EPCs.** A** Effect of GSC on the mitochondrial morphology of EPCs (5000x). The scale is 50 μm. The yellow arrows indicate mitochondria within EPCs. **B–G** Effects of GSC on the expression of the EPCs mitochondrial fusion and fission-related mRNAs Drpl (**B**), Mff (**C**), Fisl (**D**), Opal (**E**), Mfn1 (**F**) and Mfn2 (**G**).** H**-**N** Effect of GSC on the expression of mitochondrial fusion and fission-related proteins in EPCs (n = 3). Western blot analysis of Drp1 (**H**-**I**), Mff (**H**, **J**), Fisl (**H**, **K**), Opal (**H**, **L**), Mfn1 (**H**, **M**) and Mfn2 (**H**, **N**) protein expression in each group. Compared with those in the Control group, *P < 0.05, **P < 0.01, and ***P < 0.001; Compared with those in the Model group, ^#^*P* < 0.05, ^##^*P* < 0.01, and ^###^*P* < 0.001

In terms of mitochondrial function, we compared mtROS, MMP and ATP levels in senescent EPCs across the various groups following interventions with GSC and Met. The results showed that there were significant changes in mtROS, MMP, ATP between the Control and Model group after modeling. After GSC and Met intervention, there was a marked decrease in the mtROS of the GSC and Met groups compared with the Model group (Fig. 2A, B). Concurrently, there was a notable increase in the levels of ATP and MMP (Fig. 2C–E). It is noteworthy that GSC demonstrates greater efficacy than Met in enhancing mitochondrial function. Dysregulated mitochondrial dynamics are a major factor affecting mitochondrial morphology. Our results illustrated that D-gal-induced senescent EPCs leaded to excessive mitochondrial fragmentation (Fig. 3A). In contrast, GSC and Met inhibited fragmentation and restored mitochondrial morphology. Subsequently, we conducted a comprehensive assessment of the effects of GSC on mitochondrial fusion/fission through qPCR and Western blotting. D-gal leaded to a significant increase in the mRNA and protein levels of Drp1, Mff, and Fis1 in EPCs. After GSC and Met treatment, the expression of mRNAs related to mitochondrial fission was markedly lower in the GSC and Met groups than in the Model group (Fig. 3B–D). Concurrently, there was a notable decrease in the protein levels of Drp1, Mff, and Fis1 in both the GSC and Met groups (Fig. 3H–K). Although the levels of Mfn1, Mfn2, and Opa1 did not show significant changes (Fig. 3E–G and L–N), these results indicate that GSC has a similar ability to Met in preventing excessive mitochondrial fission.

### GSC regulates mitochondrial fission in D-gal-induced senescent EPCs via the AMPK pathway

**Fig. 4:**
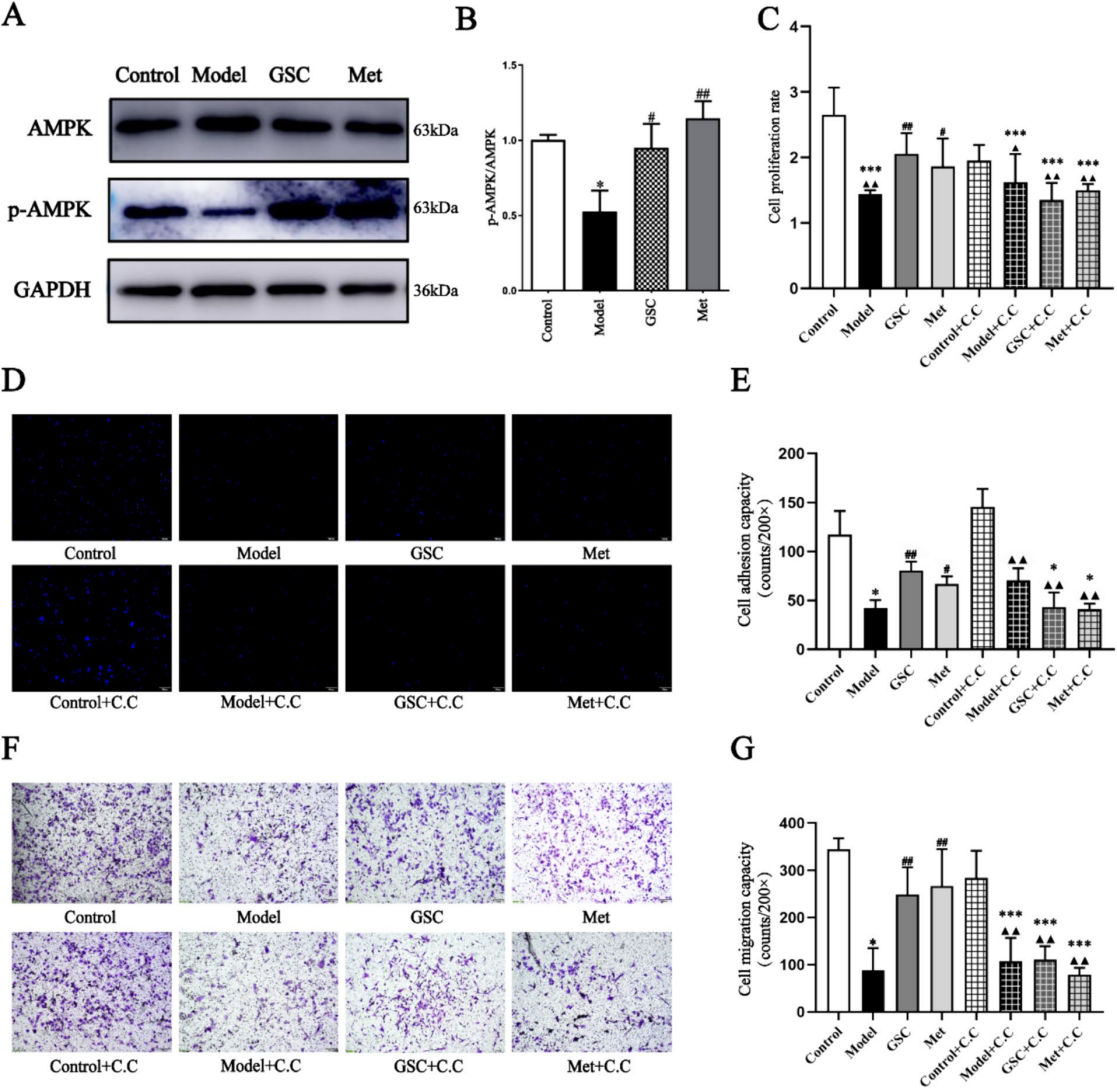
Effect of AMPK inhibitors on ECs function in each group.** A, B** Effect of GSC on AMPK and p-AMPK protein expression of EPCs (n = 3). **C** Effects of AMPK inhibitors on EPCs cell proliferation function. **D**, **E** Effects of AMPK inhibitors on cell adhesion function (200x). The scale is 50nm. **F**, **G** Effects of AMPK inhibitors on EPCs migration function (200x). The scale is 50nm. Compared with the Control group, *P < 0.05, ***P < 0.001; Compared with the Model group, ^#^*P* < 0.05, ^##^*P* < 0.01; Compared with the Control + C.C group, ^▲^*P* < 0.05, ^▲▲^*P* < 0.01

AMPK serves as a critical regulatory pathway for maintaining mitochondrial homeostasis, and it is also implicated in numerous molecular regulatory pathways during the aging process, including mitochondrial dysfunction, metabolic disorders, and cellular senescence [6]. Our study indicated that D-gal-induced aging EPCs resulted in a reduction of p-AMPK, but GSC and Met reversed this reduction (Fig. 4A, B). Consequently, we conducted an examination of the mechanisms of GSC in delaying the senescence of EPCs, specifically focusing on the AMPK pathway.

In terms of EPCs function, we conducted a comparative analysis of the impacts of GSC and Met on the proliferation and adhesion capabilities of senescent EPCs, both prior to and following the administration of AMPK inhibitors. As shown in Fig. 4C–G, GSC and Met significantly altered the reduction in both the proliferation and adhesion capabilities of D-gal-induced senescent EPCs, especially GSC. However, the enhancement of senescent EPCs function facilitated by GSC and Met is inhibited after the introduction of AMPK inhibitors. Subsequently, we investigated the alterations in mtROS, MMP, and ATP levels in senescent EPCs following the inhibition of AMPK.

**Fig. 5:**
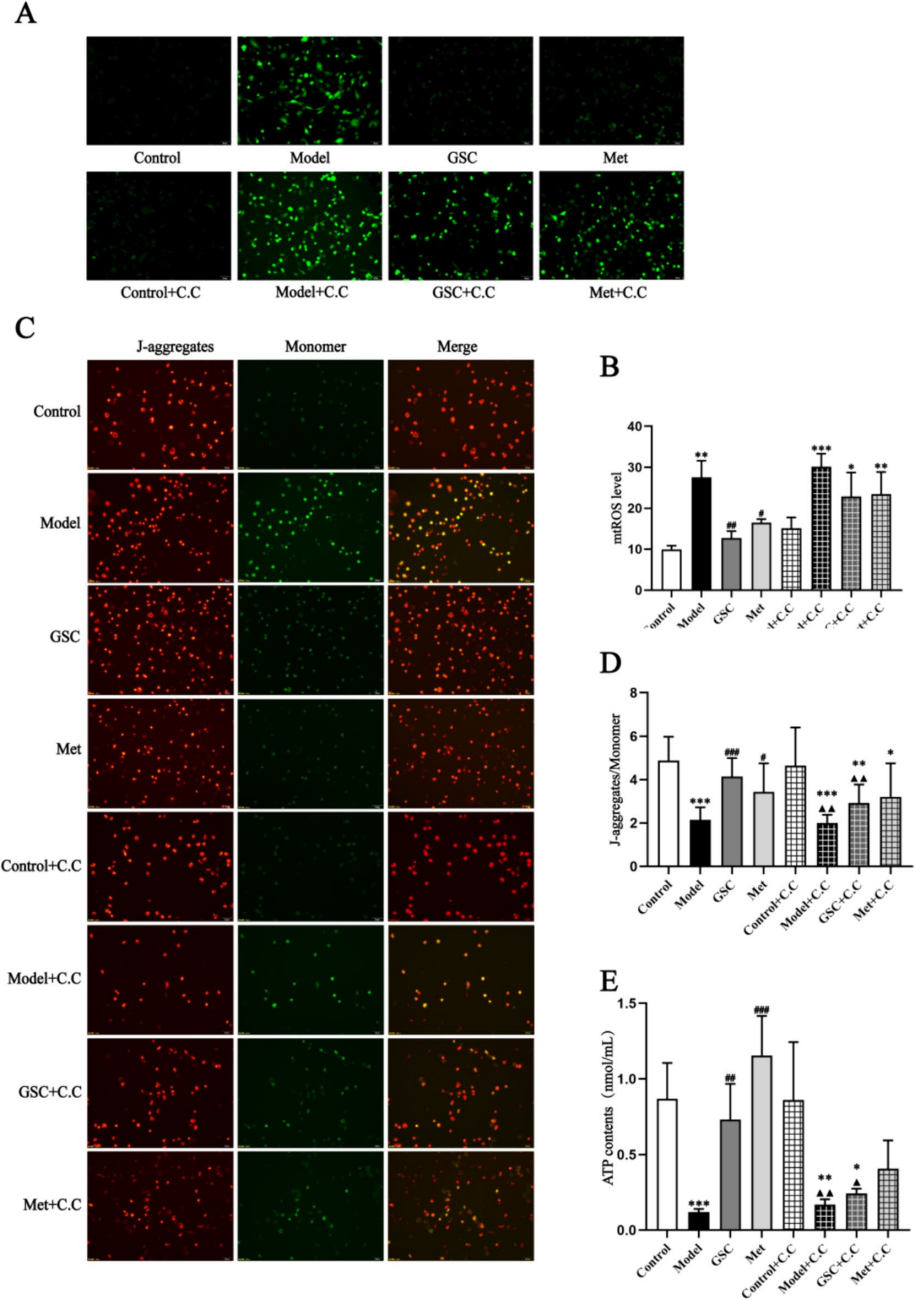
Effects of AMPK inhibitor on mitochondrial functions in each group.** A**, **B** Effects of AMPK inhibitor on mtROS level in each group (200x). The scale is 50 μm. **C**, **D** Effects of AMPK inhibitor on mitochondrial membrane potential in each group (200x). The scale is 50 μm. **E** Effects of AMPK inhibitor on ATP contents in each group. Compared with the Control group, *P < 0.05, **P < 0.01, ***P < 0.001; Compared with the Model group, ^#^*P* < 0.05, ^##^*P* < 0.01, ^###^*P* < 0.001; Compared with the Control + C.C group, ^▲^*P* < 0.05, ^▲▲^*P* < 0.01

**Fig. 6:**
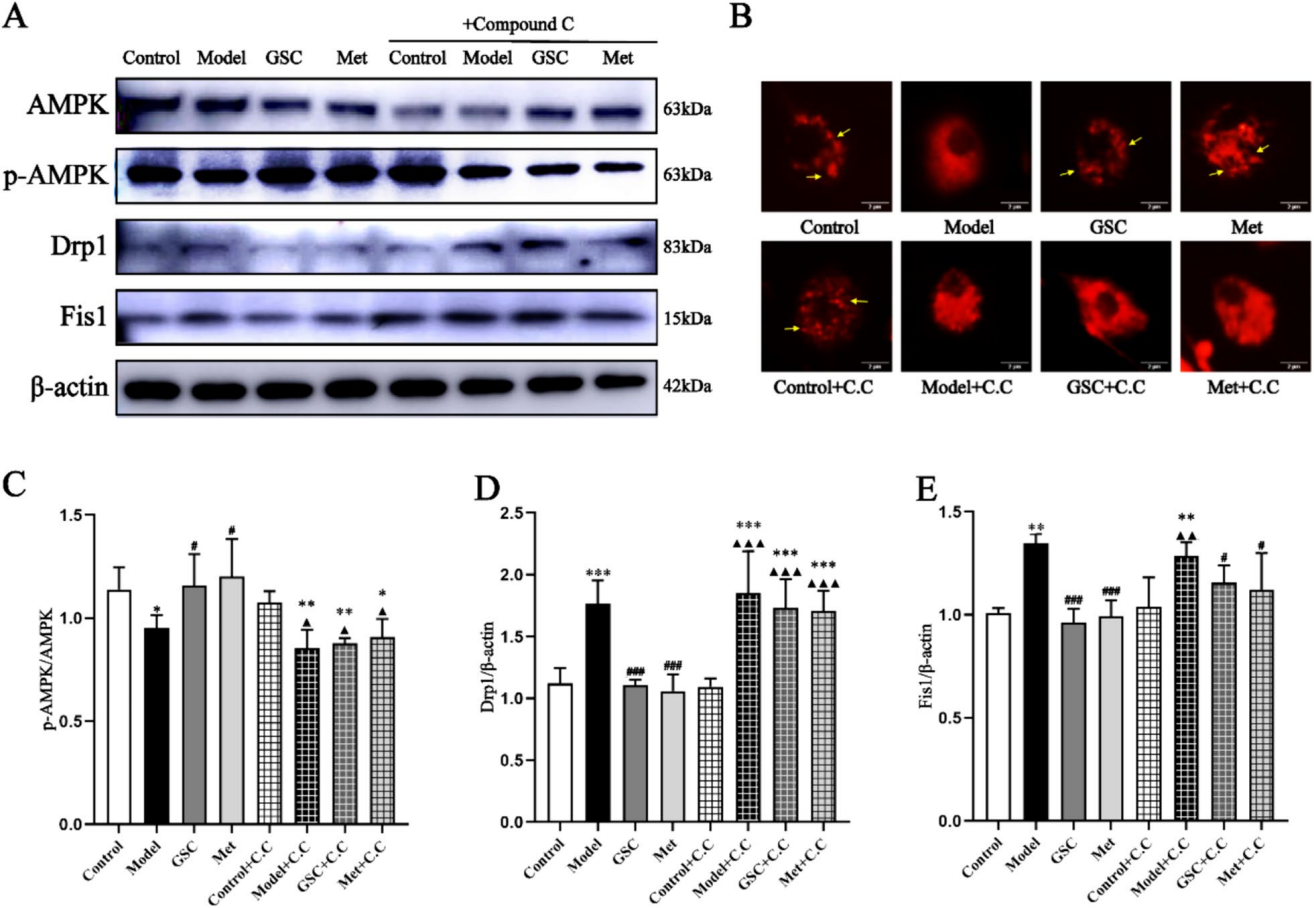
Effect of AMPK inhibitor on mitochondrial fission in each group. **A**, **C**–**E** Effects of AMPK inhibitor on AMPK pathway and mitochondrial fission proteins expression in each group (n = 3). **B** Effects of AMPK inhibitor on mitochondrial morphology in each group (5000x). The scale is 2 μm. The yellow arrows represent mitochondria within EPCs. Compared with the Control group, *P < 0.05, **P < 0.01; Compared with the Model group, ^#^*P* < 0.05, ^##^*P* < 0.01, ^###^*P* < 0.001; Compared with the Control + C.C group, ^▲^*P* < 0.05, ^▲▲^*P* < 0.01, ^▲▲▲^*P* < 0.001

**Fig. 7:**
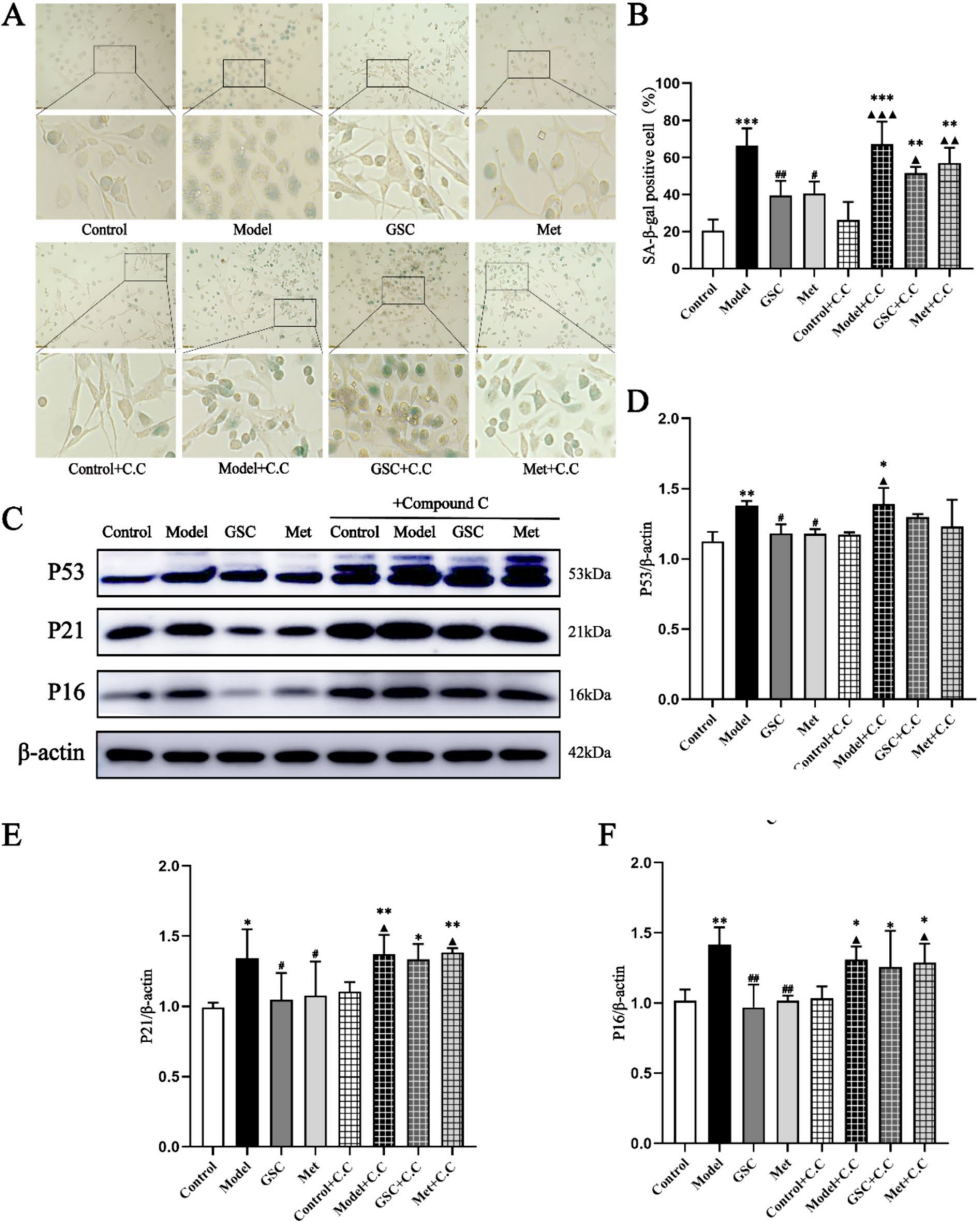
Effects of AMPK inhibitor on EPCs senescence in each group.** A**, **B** Effects of AMPK inhibitor on EPCs SA-β-gal staning in each group(200x). The scale is 50 μm.** C**–**F** Effects of AMPK inhibitor on P53 P21 and P16 proteins expression in each group (n = 3). Compared with the Control group, *P < 0.05, **P < 0.01, ***P < 0.001; Compared with the Model group, ^#^*P* < 0.05, ^##^*P* < 0.01; Compared with the Control + C.C group, ^▲^*P* < 0.05, ^▲▲^*P* < 0.01, ^▲▲▲^*P* < 0.001

The AMPK inhibitors resulted in the reversal of the beneficial effects of GSC and Met on mtROS, MMP, and ATP levels in senescent EPCs. The administration of the AMPK inhibitor led to increased levels of mtROS and a decrease in MMP and ATP levels in both the GSC and Met groups (Fig. 5). As shown in Fig. 6A and D–E, before the inhibitor was added, p-AMPK was obviously greater in the GSC and Met groups than in the Model group, and the protein expression of Drp1 and Fis1 was markedly lower. p-AMPK was decreased, and Drp1 expression was increased in the GSC + C.C and Met + C.C groups compared with those of the Control and Control + C.C groups, but there was no statistically significant difference compared with that in the Model + C.C and Model groups. However, the GSC + C.C and Met + C.C groups demonstrated an upward trend in Fis1 protein expression compared with the Model group, which was not the case for the Model + C.C group. Upon the introduction of the AMPK inhibitor, the Control + C.C group continued to display distinct elongated and short rod-like mitochondrial structures. Conversely, the Model + C.C group, the GSC + C.C group, and the Met + C.C group exhibited a diffuse staining pattern, with no discernible mitochondrial morphology evident (Fig. 6B). The most critical finding is that the intervention with the inhibitor resulted in alterations in the aging-related indicators of EPCs. Although the number of SA-β-gal staning in the GSC + C.C and Met + C.C groups were greater than those in the control and Control + C.C groups, it was not significantly different compared to the model and Model + C.C groups (Fig. 7A, B). AMPK inhibitors notably counteracted the effect of GSC and Met on diminishing the expression of P16 and P21 in senescent EPCs. Additionally, an increase in P53 expression was observed in the GSC + C.C and Met + C.C groups (Fig. 7C–F).

### EPCs from mice with D-gal-induced vascular aging improve aortic aging through autologous transplantation, and GSC enhances the effect of transplantation

Autologous EPCs transplantation and mobilization of EPCs by drugs contribute to vascular repair and neovascularization, but the number and functional activity of EPCs is impaired by aging, and the efficacy of transplantation is not satisfactory. In this segment, we concentrated on examining the influence of GSC intervention on the transplantation effect and efficacy of EPCs.

**Fig. 8:**
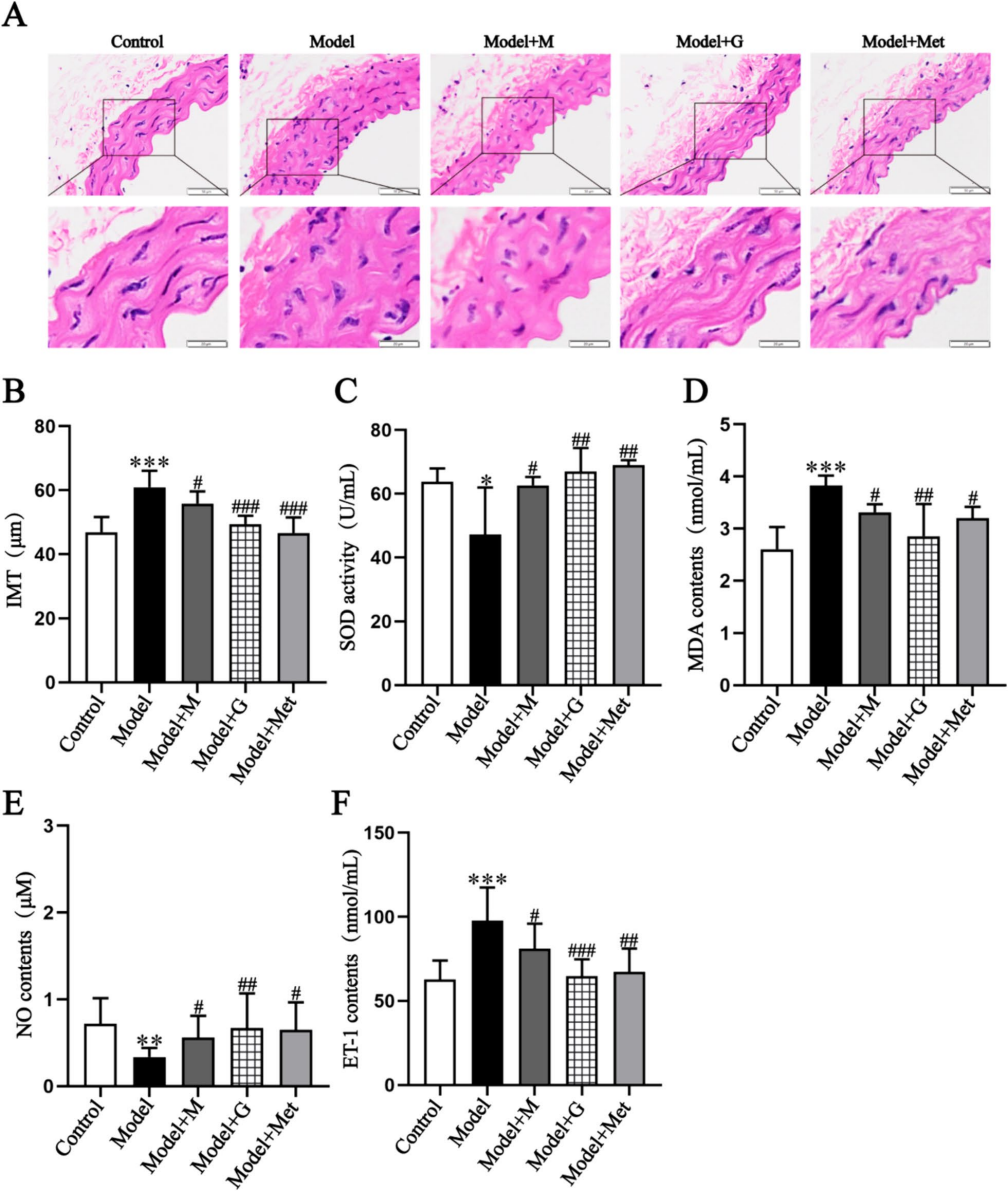
Comparison of pathological changes, oxidative stress and vascular endothelial function of mouse in each group.** A**, **B** The HE staining and intima-media thickness of mice aorta (200x), The scale is 50 μm.** C**, **D** Comparison of SOD activity and MDA contents in serum of mice in each group (n = 6). **E**, **F** Comparison of NO and ET-1 contents in serum of mice (n = 6). Compared with the Control group, ^*^P < 0.05, ^**^P < 0.01, ^***^P < 0.001; Compared with the Model group, ^#^*P* < 0.05, ^##^*P* < 0.01, ^###^*P* < 0.001

**Fig. 9:**
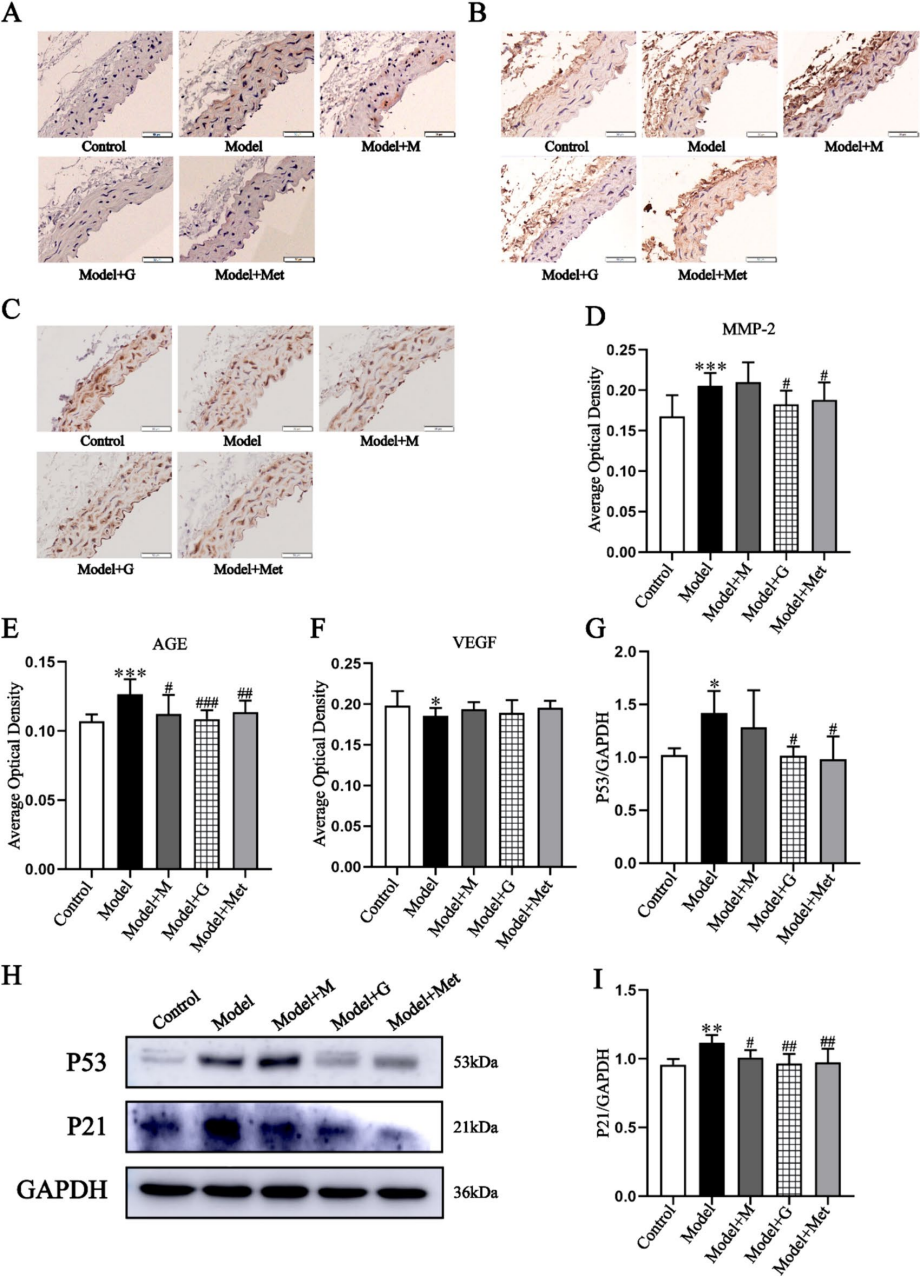
Comparison of AGEs accumulation, collagen elastin fiber damage, angiogenic function and the expression of aging-related proteins in aorta of mice.** A**, **E** The AGEs expression results of mice aorta (IHC, 200x, 50μm). **B**, **D** The MMP-2 expression results in mice aorta (IHC, 200x, 50μm). **C**, **F** The VEGF expression results in mice aorta (IHC, 200x, 50μm). **G–I** The western bolt analysis of P53 and Pp21 (n = 3). Compared with the Control group, ^*^P < 0.05, ^**^P < 0.01, ^***^P < 0.001; Compared with the Model group, ^#^*P* < 0.05, ^##^*P* < 0.01, ^###^*P* < 0.001

According to the description of 2.2, mice were subjected to the transplantation of EPCs via the tail vein. Based on the results of laboratory manipulation and In Vivo Imaging System (**Supplementary Fig. 11**). The final transplantation results were as follows: The numbers of successful tail vein injections in the model group, Model + M group, Model + G group, and Model + Met group were 10, 8, 9, and 10. The results indicate that GSC enhances the effect of EPCs transplantation on improving mouse aortic morphology and IMT. There is a marked disorganization of the aortic structure in Model and Model + M groups, but it in Model + G and Model + Met groups mproved the situation (Fig. 8A). The IMT results showed that the Model + G group and Model + Met group had lower IMTs than did the Model group (Fig. 8B). After drug intervention in EPCs transplantation, the SOD activity increased and MDA decreased in the Model + G and Model + M groups. GSC demonstrated superior efficacy compared to Met in enhancing endothelial function, NO levels were significantly increased and ET-1 levels were significantly decreased in Model + M and Model + G groups (Fig. 8E). At the same time, both Model + G and Mede + M can reduce matrix metalloproteinase-2 (MMP-2) and advanced glycation end products (AGEs), but had no effect on VEGF (Fig. 9A–F). The results indicated that GSC and Met can improve vascular stiffness and alleviate collagen fiber damage. In terms of vascular aging levels, both EPCs treated with GSC and Met showed better effects in delaying aging. The levels of P53 and P21 in groups Model + M and Model + G both significantly decreased,

## Discussion

CVDs are the leading cause of aging-related health problems worldwide [1], and aging is the largest risk factor for CVD [2]. EPCs transplantation has been instrumental in regulating endothelial function, ischemic tissue and vascular proliferation in patients with CVD [34]. However, there are currently controversial issues related to the effectiveness of EPCs in terms of differentiation and migration [35]. We previously reported that GSC inhibits mitochondrial autophagy through the AMPK pathway to play a role in delaying vascular senescence in diabetic mice [15].

We selected D-gal as the modeling drug for the aging model because D-gal can accelerate aging in animals by increasing MDA levels, decreasing SOD activity, and leading to oxidative stress [36]. In D-gal-induced senescent rats, mitochondrial dysfunction results in time-dependent degeneration, imbalanced mitochondrial kinetic processes, and increased autophagy and apoptosis [40]. The results of our experiments also demonstrated that GSC and Met ameliorate mitochondrial dysfunction, as indicated by elevated mtROS levels and reduced ATP and membrane potential in D-gal-induced senescent EPCs (**Supplementary Fig. 7**).

Vascular aging manifests as vascular structural changes and endothelial dysfunction. Changes in the vascular structure are closely related to age. The media and intima are altered, which leads to thickening of the vessel wall and an increase in the IMT, and these are also observed during the aging process [42]. In terms of endothelial dysfunction, the imbalance between vasodilation and constriction is an important factor affecting endothelial function. The synthesis and release of vasodilators and vasoconstrictors, such as NO and ET-1, regulate the balance between vasodilation and vasoconstriction [43, 44]. Met is a clinical drug used to treat type 2 diabetes mellitus, but it is also effective at slowing aging and reducing aging-related diseases [45]. It is now established that Met exerts antiaging effects by affecting the AMPK pathway, ROS levels, protein homeostasis, telomere shortening, and genetic expression [46, 47]. Therefore, we chose Met as the positive control drug for this experiment.

Mitochondrial dynamics shape the mitochondrial network, which contributes to mitochondrial function and quality control [51]. Mitochondrial dynamics include the processes of mitochondrial biosynthesis, fusion, and fission [52]. The dynamic equilibrium of mitochondrial fusion/fission plays a crucial role in the maintenance of mitochondrial function. Mitochondrial fusion is mediated by Mfn and Opa1, which are key in regulating the fusion of inner and outer mitochondrial membranes and endosomes [53]. Mitochondrial fission is regulated mainly by Drp1 and is accomplished through the recruitment of the mitochondrial kinetic proteins 45 and 53, Mff, and Fis1 [54, 55]. An imbalance in mitochondrial dynamics occurs when organisms and cells age. However, there is controversy about the regulation of aging by mitochondrial fusion/fission proteins. Several studies have indicated that mitochondrial fission dysfunction occurs in senescent cells and tissue and cellular function can be improved by promoting the expression of mitochondrial fission-related proteins such as Drp1 [56–58]. However, there have also been studies that show diametrically opposite results. Senescence induces a shift in mitochondrial dynamics toward fission, which in turn leads to disturbances in cellular metabolism [59, 60], and the cognitive dysfunction of rats with D-gal-induced aging is significant when mitochondrial fission-related proteins such as Drp1 are inhibited [61].

According to our experimental results, D-gal-induced senescence of EPCs causes excessive mitochondrial fragmentation, resulting in elevated expression of the Drpl, Mff and Fis1 proteins and mRNAs, whereas GSC and Met ameliorate excessive mitochondrial fragmentation in senescent EPCs (Fig. 3). This result is not consistent with those of some of the studies regarding EPCs [62], possibly because of differences in the source of the senescent cells, type of cells, modeling time and drug dose. The dynamic equilibrium of mitochondrial fusion has not yet been clearly established, and contradictory results occur even in different tissues of the same individual. Qingyang Li et al. showed that the expression of Drp1 is significantly decreased in the liver of D-gal-induced aging mice but is increased in the skin and small intestine compared with that in the control group [63]. Therefore, it is necessary to study the dynamic equilibrium of mitochondrial fission and fusion. Interestingly, the results of this study revealed that there was no significant difference between the Control and Model groups and that the expression of the Mfn1 mRNA was significantly greater in the GSC group than in the other groups (Fig. 3F, M). We speculate that this difference may be related to the main components of GSC, ginsenoside Rg_3_ and total saponins of *Panax notoginseng*.

The regulation of mitochondrial fission is closely related to the AMPK pathway, but the regulation of AMPK is bidirectional. NR4A1 promotes TNF-α-induced chondrocyte death and migration injury through activation of the AMPK/Drpl/mitochondrial fission pathway [64]. Another study revealed that activation of AMPK induces mitochondrial fragmentation [65]. The above studies illustrate that the AMPK pathway promotes mitochondrial fission. However, Wiren Chen et al. reported that melatonin attenuates vascular calcification by inhibiting mitochondrial fission through activation of the AMPK/Drpl signaling pathway [26]. Miaomiao Hao et al. showed that resveratrol inhibits the TNF-α-induced decrease in the MMP by activating AMPK and decreasing Drpl activity [66]. These studies suggest that activation of AMPK inhibits mitochondrial fission. However, the results of our study showed that when the AMPK pathway was inhibited, GSC and Met were unable to improve the ROS activity and MMP in D-gal-induced senescent EPCs (Fig. 5A–D). At the same time, GSC and Met significantly reduced Drp1-mediated mitochondrial fission (Fig. 6A). The above results may be due to EPCs were extracted from mouse bone marrow and an aging model. It is important that GSC is more effective than Met in improving mitochondrial function and enhancing the antioxidant capacity of EPCs. The intervention of GSC in aging EPCs shows better results in mtROS, mitochondrial membrane potential, and ATP levels compared to the Met group (Fig. 2). At the same time, the cell adhesion function of the GSC group is also better than that of the Met group (Fig. 4D, E).

**Fig. 10:**
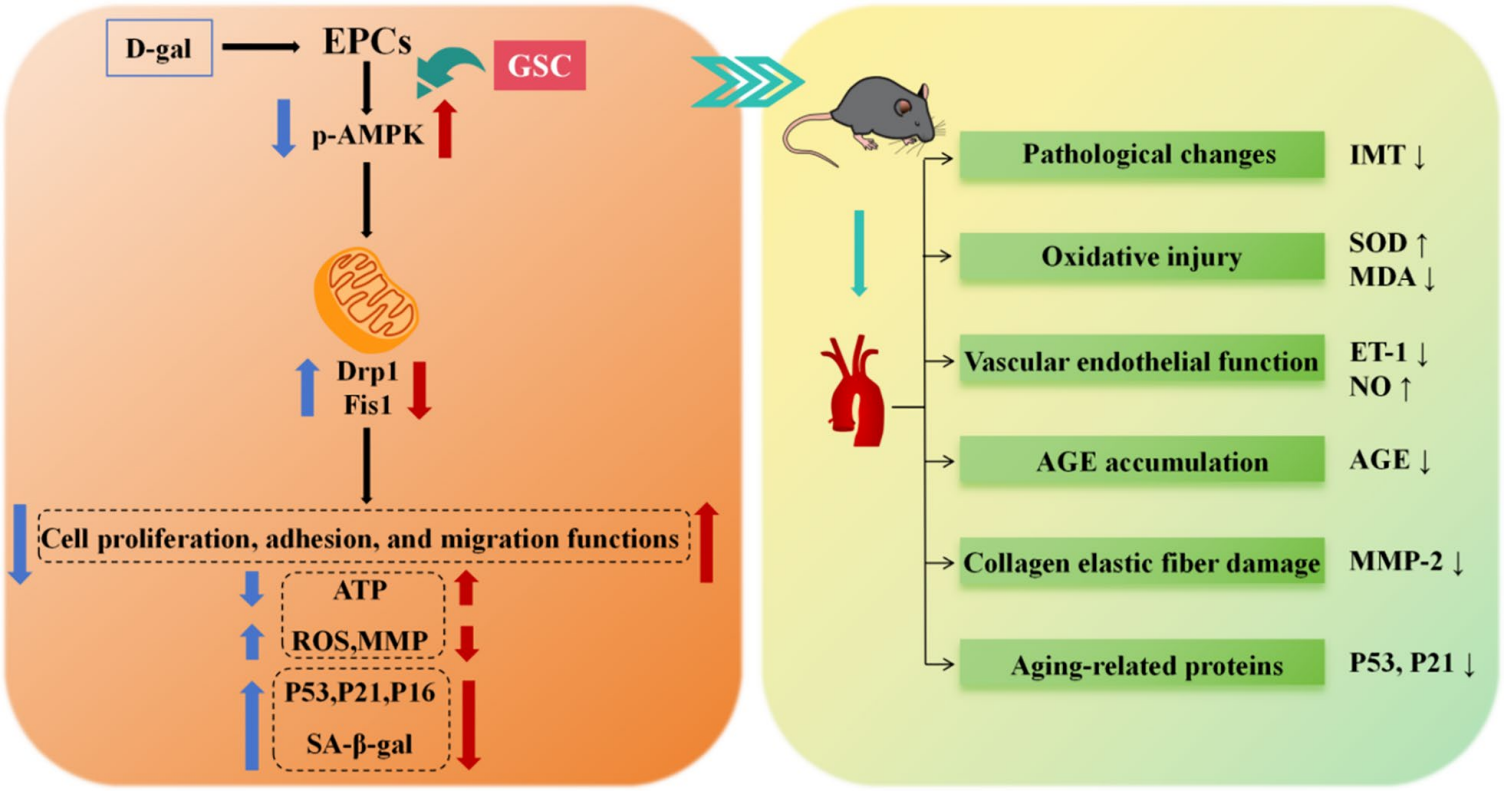
Mechanism of GSC intervention on D-gal-induced senescent EPCs and optimization of autotransplantation efficacy in vascular aging mice. GSC inhibited mitochondrial fission by activating AMPK phosphorylation, which in turn improved the cellular function, mitochondrial function and senescence phenotype of D-gal-induced senescent EPCs. In addition, in vitro intervention in EPCs promoted improvements in aortic pathology, oxidative stress, vascular endothelial function, AGEs accumulation, collagen-related elastin fiber damage and aging-related proteins in EPCs autografts

EPCs are precursor cells of ECs that repair damaged areas and differentiate into ECs, and EPCs play an important role in vascular repair and neogenesis. Postnatal neovascularization is not only dependent on mature ECs sprouting in preexisting vessels but also involves the contribution of circulating EPCs of bone marrow origin. Circulating adult EPCs can nest to ischemic tissues and thus aid in cardiovascular generation [67]. EPCs are categorized into two distinct subgroups: early-growth and late-growth [68]. During cell culture, early-growth EPCs appeared within 4–7 days, and late-growth EPCs appeared after 14–21 days. Early and late EPCs have synergistic effects on vascular repair and regeneration. Early EPCs secrete growth factors such as VEGF, SDF-1, NO, and vascular endothelial leukocyte hormone-8 to promote endothelial repair through a paracrine mechanism [69–71]. Late EPCs, which are morphologically similar to EPCs, have strong proliferative potential and can form functional blood vessels in vivo [72]. These two types of EPCs are used at different times to repair endothelial damage caused by vascular aging. It has been demonstrated that transplantation therapy with EPCs is an important endogenous vascular repair strategy that can be used as a treatment for diseases associated with endothelial integrity and dysfunction. EPCs from young bone marrow have been shown to reverse age-related impairment of cardiac angiogenesis [73]. However, EPCs are also characterized by low numbers in the body, immune rejection, and susceptibility to microenvironmental influences, and these factors have led to unsatisfactory transplantation results. Relevant clinical studies have shown a correlation between the number of surviving EPCs after transplantation and plasma SOD and MDA levels [74]. The results of the present study indicated that autologous transplantation of senescent EPCs significantly ameliorated IMT, SOD activity, and MDA, NO and ET-1 levels in D-gal-induced vascular aging mice. Therefore, GSC improves the antioxidant capacity of the body and the microenvironment after transplantation of EPCs, and this outcome favors the survival of EPCs and enhances the efficacy of transplantation therapy. Moreover, autografts also decreased the protein expression of AGEs, MMP-2 and P21. Treatment of EPCs with GSC and Met in vitro improved the above indices and reduced the protein expression of P53. The above results suggest that autograft EPCs are beneficial for treating vascular aging and that GSC and Met interventions enhance the efficacy of autografts for treating EPCs in vitro (Fig. 10). The results of transplantation experiments showed that GSC could significantly increase MDA level and enhance the antioxidant capacity of aging mice compared with Met (Fig. 8D). At the same time, the improvement of AGEs and MMP-2 in Model + G group suggests that it is more effective in delaying the vascular stiffness in aging mice (Fig. 9A, B and D–E).

As shown in Fig. 10, this study determined that GSC can ameliorate D-gal-induced senescence in EPCs by inhibiting Drpl-mediated mitochondrial fission and enhance the transplantation efficiency of EPCs in vascular aging of mice. The AMPK pathway is a key mechanism for the resolution of mitochondrial fission. The results of this study strongly support further exploration of the feasibility of transplanting EPCs and GSC intervention for vascular aging-related diseases. In vitro autografting of EPCs after GSC intervention may be a promising pathway for treating advanced aortic senescence and CVD.

## Limitations of the study

There are several limitations in our study. There may be some differences between D-gal-induced senescent mice and senescent individuals, which need to be validated in the clinic. The transplantation protocol can be further explored. Future transplantation studies will need to be optimized for different time points, different numbers of cells transplanted, and combinations of multiple interventions with drugs. Nevertheless, our study provides new ideas for stem cell therapy to combat vascular aging and CVD.

## Supplementary Information

Additional file 1.

## Data Availability

Data will be made available on request, please contact the corresponding author.

## Abbreviations

ATP: Adenosine triphosphate
AMPK: Adenosine 5 ‘-monophosphate-activated protein kinase
CVD: Cardiovascular disease
D-gal: D-galactose
ECs: Endothelial cells
EPCs: Endothelial progenitor cells
H&E: Hematoxylin and eosin
IHC: Immunohistochemical
IMT: Intima-media thickness
MDA: Malondialdehyde
mRNAs: Messenger Ribonucleic Acids
Met: Metformin
MMP: Mitochondrial membrane potential
mtROS: Mitochondrial reactive oxygen species
ROS: Reactive oxygen species
SOD: Superoxide dismutase
VEGF: Vascular endothelial growth factor
GSC: Yiqihuoxue decoction
