# Supplementary material for: Yiqihuoxue decoction (GSC) inhibits mitochondrial fission through the AMPK pathway to ameliorate EPCs senescence and optimize vascular aging transplantation regimens
Source: Chin Med. 2024 Oct 14;19:143. doi: 10.1186/s13020-024-01008-7 (PMC11479513; doi:10.1186/s13020-024-01008-7)
Supplement: Supplementary file 1 — Additional file 1. [file 13020_2024_1008_MOESM1_ESM.docx]

Supplementary

# Materials and methods

## Materials and reagents

According to the results of our previous study [12], GSC is composed of ginseng (*Panax ginseng* C.A.Mey.), San Qi (*Panax notoginseng* (Burk.) F.H.Chen.) and Chuan Xiong (*Ligusticum sinense 'Chuanxiong'* *Hort.*). The medicinal materials used were purchased from the Beijing Tong Ren Tang Group in China, and the drug lot numbers used were as follows: ginseng (Jilin, YL-302-1403-001), San Qi (Yunnan YL-201-1403-001) and Chuan Xiong (Sichuan YL-018-1403-001). All medicinal materials were identified by the Institute of Chinese Materia Medicine, China Academy of Chinese Medical Sciences. The three herbs were mixed in certain proportions (2:3:4) and crushed. Pure water and 95% ethanol were used as extraction solvents (1:10 ratio), and GSC was extracted twice by heating at 90 °C and 70 °C for 2 h at boiling. The chemical contents of GSC were determined using UPLC-LTQ-Orbitrap/MS and UPLC-Q-TOF/MSE mass spectrometry. According to the standards of the *Pharmacopoeia of the People's Republic of China* (2020), the content of the hallmark ingredients in GSC was determined using HPLC (Supplementary **Figs.** 1-5, **Table 1**-5). Metformin (Met) hydrochloride (serial number: D9351) was obtained from Solarbio & Science Technology (Beijing) Co., Ltd. D-galactose (serial number: G0750-100G) was purchased from US Sigma‒Aldrich.

## Mitochondrial reactive oxygen species (mtROS) and the mitochondrial membrane potential (MMP)

EPCs were seeded in 6-well plates (1 × 10^4^ cells/well). When the drug intervention was complete, the medium was aspirated, and the cell surface was washed 3 times with PBS. According to the manufacturer's instructions (Beyotime, China, S0033M), the 6-well plates were filled with 1 mL of DCFH-DA (1:1000) and incubated at 37 °C in an incubator for 20–30 min in the dark. The dilutions were aspirated, the serum-free medium was used to wash the cells three times, and the cells were placed under a fluorescence microscope (OLMPUS, Japan, BX61VS) for observation. At least 5 fields of view were photographed per group.

MMP detection was performed as previously described [18]. The staining working solution and JC-1 staining buffer (1×) were prepared according to the product manual (Beyotime, China, C2006). Equal volumes of complete medium and staining solution were added, and the cells were incubated in an incubator for 20 min. After incubation, the cells were washed twice with JC-1 staining buffer (1×). The fluorescence staining of the monomers was observed under a fluorescence microscope at an excitation wavelength of 490 nm and an emission wavelength of 530 nm. The fluorescence staining of the polymers (J-aggregates) was observed at an excitation wavelength of 525 nm and an emission wavelength of 590 nm. At least five fields of view were selected from each group.

## Isolation and culture of bone marrow-derived EPCs

EPCs were derived from the bilateral femurs and tibias of male C57BL/6N mice (6 weeks of age) by aseptic means, as previously reported [18, 19]. After the mice were euthanized, the epiphyses of the tibia and fibula were separated, and the mouse diaphysis was placed in 1% double-antibody EGM-2MV culture medium. Afterward, the bone marrow cavity was flushed using culture medium. The cells were inoculated in T25 vials at a density of 1 × 10^7^/ml. The solution was changed every half day for the first 4 days, after which the solution was changed every 3 days. Subsequent experiments were performed after seven days of induction culture. Cultured EPCs were characterized by the expression of CD34 (Invitrogen, USA, 13-0341-82), CD133 (Invitrogen, USA, 12-1331-80) and CD309 (Invitrogen, USA, 13-5821-81) by confocal microscopy (Olympus, Japan, FV1000)[20].

## Adenosine triphosphate (ATP) detection

Total ATP levels were measured by chemiluminescence using an ATP content test kit according to the instructions (Nanjing Jiancheng Bioengineering Institute, China, A095-2-1). Briefly, the EPCs were placed on ice for lysis and centrifugation. Then, 100 μl of enzyme working solution was added to a 96-well white plate and incubated for 5 min at room temperature. Afterward, 10 μl of standard or sample was added to each well, and the chemiluminescence values were determined by a luminometer; ATP concentrations were determined using a BCA protein concentration kit and converted to nmol/mg.

## SOD, MDA, NO and ET-1 tests

Blood was collected retro-orbitally. After clotting at room temperature, the serum samples were obtained by centrifugation at 3500 rpm for 15 min and stored at −80 °C until further analysis. Total SOD, MDA, NO and ET-1 detection kits (Nanjing Jiancheng Bioengineering Institute, China; A001-3, A003-1, A013-2-1, H093) were used to determine the MDA, NO and ET-1 levels and SOD activity. The content of the reagent was determined and calculated in strict accordance with the manufacturer’s instructions.

## Cell proliferation assay

As described previously[24], EPCs were seeded in 96-well plates (1 × 10^4^ cells/well). Then, 10 μl of Cell Counting Kit-8 reagent (CCK-8; Dojindo, Japan, LG615) was added to each well, and the plates were incubated at 37 °C in the dark for 2-3 h. The absorbance at 450 nm was measured using an automatic microplate reader (BioTek, USA, Synergy H1).

## Cell migration assay

The migration of the EPCs was evaluated using Transwell assays as previously reported[25]. Briefly, the concentration of EPCs was adjusted to 2×10^5^ cells/ml using serum-free medium, 100 μl of cell suspension was added to the upper chamber of the Transwell, and 600 μl of complete medium was added to the lower chamber. The cells were cultured in an incubator for 24 h. Afterward, the small chamber of the Transwell was fixed, stained with crystal violet, washed, and air-dried. A minimum of 5 fields of view were taken under the microscope for each group, and the number of cells in each field of view was counted separately to determine the migration ability of the cells.

## Cell adhesion assay

EPCs were inoculated in 12-well plates coated with fibronectin at a density of 2×10^5^ cells/ml according to previously reported methods[26, 27]. After incubating for 3 h, the unattached cells were washed away with PBS, fixed by adding 4% paraformaldehyde at room temperature for 10 min, and stained by the dropwise addition of 4’,6-diamidino-2-phenylindole (DAPI) for 10 min. After washing with PBS, the 12-well plates were placed under a fluorescence microscope, and at least 5 fields of view were randomly selected for observation and counting to determine cell adhesion ability.

## SA-β-gal cell senescence staining

To assess the senescence of EPCs, SA-β-Gal activity was measured as previously described[28]. According to the instructions for SA-β-gal cell senescence staining (GENMED Scientific, Inc., USA; GMS10012.1), EPCs were seeded in 24-well plates (2 × 10^4^ cells/well), and reagents A, B and C were added for washing, fixation and acidification. Afterward, preheated staining working solution was added, and the plates were incubated in a CO_2_-free, 37 °C incubator for 16 h. The plates were placed under a light microscope with at least 5 fields of view in each group. The total number of cells in the field of view and the number of blue-stained cells were counted separately to calculate the percentage of blue-stained cells.

# Figure and Table

**Fig. 1** | HPLC spectra of Ginsenoside Rg1, Ginsenoside Re and Ginsenoside Rb1 in Ginseng.

**Table1** Determination results of indicator components in Ginseng

| Component | Content（%） |
| --- | --- |
| GinsenosideRg1 | 0.23 |
| Ginsenoside Re | 0.28 |
| Ginsenoside Rb1 | 0.37 |

**Fig. 2** | HPLC spectra of Ginsenoside Rg1, Ginsenoside Rb1 and Panax notoginseng saponin R1.in Panax notoginseng

**Table 2** Content determination results of each indicator component in Panax notoginseng (Burk.) F. H.Chen

| Component | Content（%） |
| --- | --- |
| notoginsenoside R1 | 1.09 |
| Ginsenoside Rg1 | 5.54 |
| Ginsenoside Rb1 | 3.54 |

**Fig. 3** | HPLC spectrum of ferulic acid in Ligusticum chuanxiong

**Table 3** Determination results of indicator components in Ligusticum chuanxion

| Component | Content（%） |
| --- | --- |
| ferulic acid | 0.22 |

**Fig. 4** | HPLC spectrum of ferulic acid in GSC

**Fig. 5** | HPLC spectra of notoginsenoside R1, ginsenoside Rg1, ginsenoside Re and ginsenoside RB1 in GSC

**Table 4** Determination results of components in panax notoginseng chuanxiong extract

| Component | Content（%） |
| --- | --- |
| ferulic acid | 1.00 |
| notoginsenoside R1  Ginsenoside Rg1  Ginsenoside Re  Ginsenoside Rb1 | 8.45  55.84  6.47  44.57 |

### Culture and identification of EPCs

Mouse bone marrow mononuclear cells were induced in culture until day 4, at which point most of the adherent cells were ovoid. Early EPC morphology changed from ovoid to shuttle in shape after 7 days of induction culture; after 20 days of induction culture, the late EPCs exhibited cobblestone-like changes, as shown in **Supplementary Fig. 6A**. The immunofluorescence results showed that the EPCs were positive for CD34, CD133 and CD309 expression (**Supplementary Fig. 6B**). These results show that the cells extracted and induced in this experiment were EPCs.

3.1.2 Effect of different concentrations of D-gal on the activity and cell staining of EPCs

We first observed the effect of different concentrations of D-gal on EPC viability and senescence. After 24 h of drug intervention, the D-gal concentration was determined via SA-β-gal staining and CCK-8 assay. An increase in D-gal concentration decreased cell viability. When the concentration of D-gal was 10 g/L, the cell viability was significantly greater than that in the control group (*P* < 0.05). However, concentrations of 20 g/L, 40 g/L, and 80 g/L significantly reduced the percentage of viable EPCs (*P* < 0.001). After 24 h of D-gal intervention at concentrations of 40 g/L and 80 g/L, the viability of the EPCs decreased to less than 50%. The SA-β-gal staining results showed that 5 g/L, 10 g/L, 20 g/L, 40 g/L, and 80 g/L D-gal caused the percentage of blue-stained EPCs to increase (*P* < 0.001). The 20 g/L concentration resulted in the highest percentage of blue staining. Therefore, approximately 70% of the cells were considered active, and the 20 g/L concentration had the highest percentage of senescent cells (**Supplementary Fig. 7**).


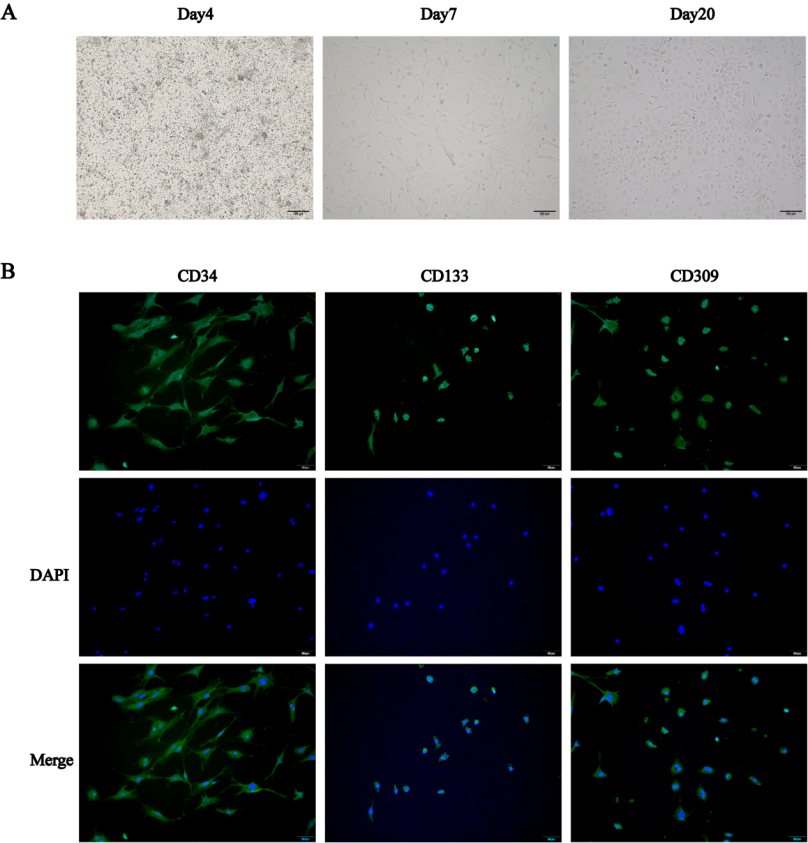


**Fig. 6** | **Culture and identification of EPCs in mouse bone marrow. A** Representative micrographs of cell clusters on day 4, early EPCs on day 7 and late EPCs generated on day 20 of mouse bone marrow cell culture(100x), with a scale of 100μm. **B** The specific markers CD34, CD133 and CD309(200x), with a scale of 50μm.


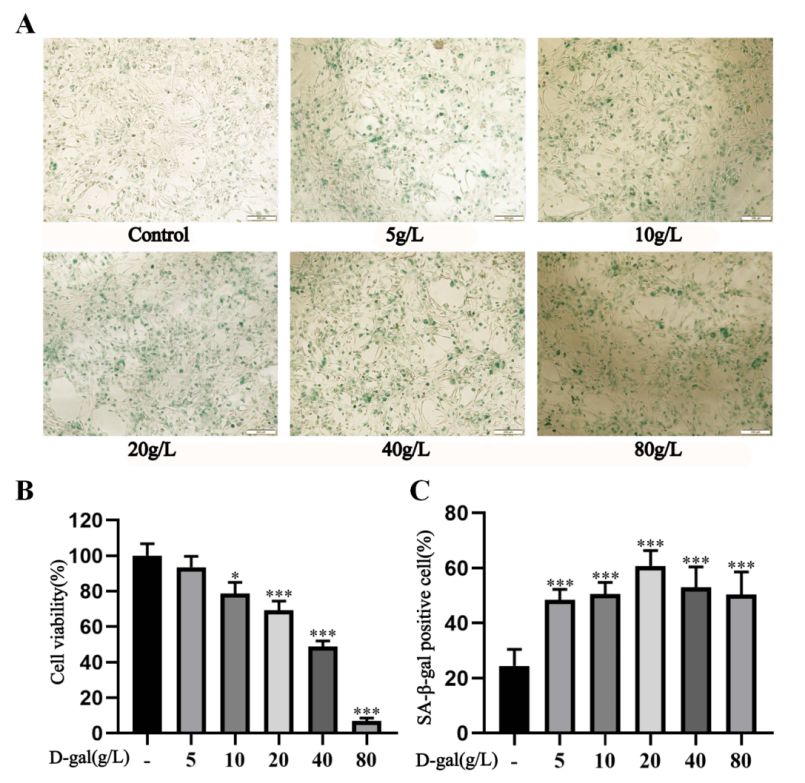


**Fig. 7** | **Effect of different concentrations of D-Gal on cell viability and cell senescence staining (200x).** **A** SA-β-gal staining of different concentrations of D-gal intervention for 24 hours (200x), with a scale of 50μm. **B** Intervention of different concentrations of D-gal on cell viability for 24 hours. **C** Effect of Different Concentrations of D-gal on the staining ratio of senescent cell after 24 hours of Intervention. Compared with the Control group, **P*<0.05, ****P*<0.001.

### Effects of different concentrations of GSC on the viability of D-gal-induced senescent EPCs and on cellular senescence

Next, we selected the optimal drug concentration of GSC through 24 h of combined treatment with D-gal and different concentrations of GSC. The results of cell viability and cell senescence staining were evaluated by CCK-8 and SA-β-gal assays. The cell viability rate of the model group was significantly lower than that of the control group (P < 0.001). The cell viability rate tended to increase with increasing concentration of GSC. However, there was no statistically significant difference in the cell viability at 25 mg/L, 50 mg/L or 100 mg/L compared to that of the model group. When the concentration of GSC was increased to 200 mg/L, the cell viability was significantly greater than that in the model group (P < 0.01). At a concentration of 400 mg/L, there was a significant difference in the cell viability compared to that of the model group (P < 0.05). However, when the concentration was increased to 800 mg/L, the cell viability decreased, but the difference was not statistically significant. The percentage of cells in the control group was significantly lower than that in the model group (P < 0.001). Similarly, the percentage of SA-β-gal-stained cells in the GSC group was significantly lower than that in the model group (P < 0.01); however, the percentage of blue-stained cells was the lowest at 800 mg/L, followed by 200 mg/L and 400 mg/L. Therefore, 200 mg/L was selected as the concentration of GSC in this experiment (**Supplementary Fig. 8**).


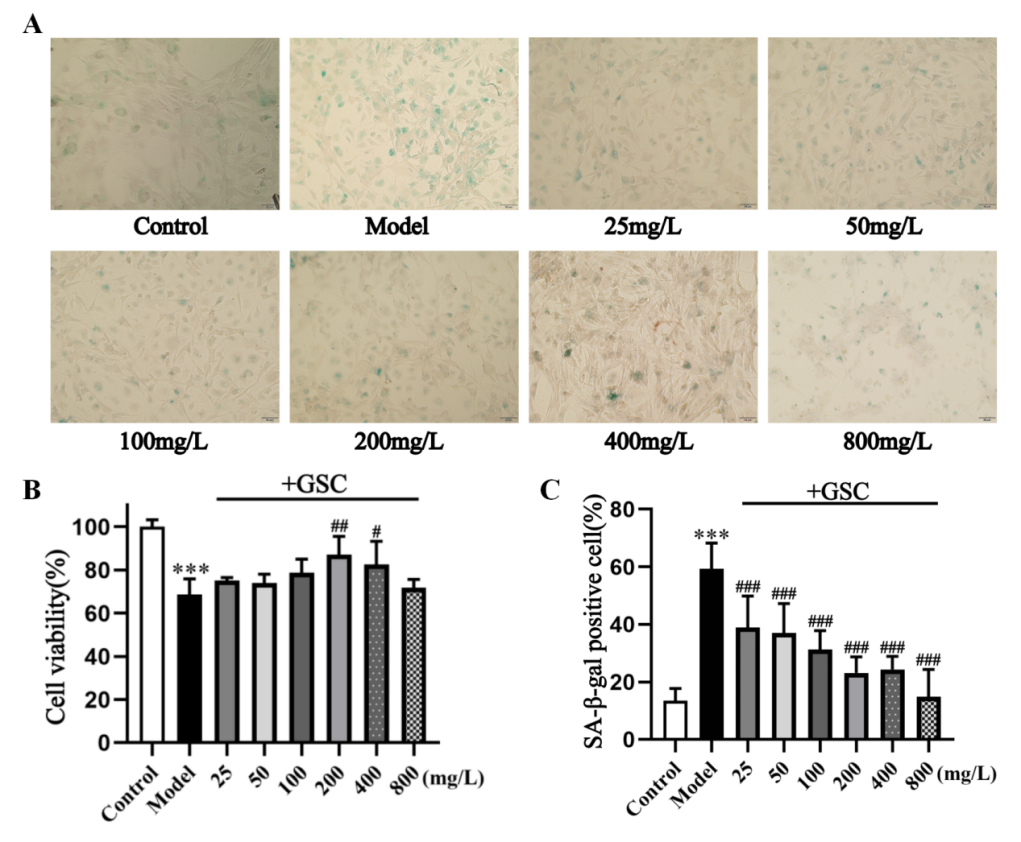


**Fig. 8** | **Effects of different concentrations of GSC on cell viability and Cell senescence staining.** **A** The SA-β-gal staining of different concentrations of GSC intervention for 24 hours (200x, scale at 50μm). **B** Cell viability rate of different concentrations of GSC intervention for 24 hours. **C** Different concentrations of GSC intervene in the staining ratio of cell senescence for 24 hours. Compared with the Control group, ****P*<0.001; Compared with the Model group, **P*<0.05, ***P*<0.01, ****P*<0.001.

### Effects of Met on the Activity of D-gal-Induced Senescent EPCs and Staining for Determination of Cellular Senescence

We used the same method to determine the ideal concentration of Met through CCK-8 assays and SA-β-gal staining. After 24 h of intervention, the cell viability of the Met group was significantly lower than that of the control group. The cell viability of the 0.5 mM and 1 mM groups increased compared to that of the model group (*P* < 0.05, *P* < 0.01). Importantly, the cell viability of the 2 mM, 4 mM, and 8 mM concentration groups apparently increased (*P* < 0.001), and the 8 mM concentration of Met had the greatest effect on cell viability. However, cell viability tended to decrease as the Met concentration continued to increase. The percentage of SA-β-gal-positive cells in the control group was significantly lower than that in the model group (*P* < 0.001). The other groups displayed the same results (*P* < 0.001). In summary, we used the same drug selection method that we used to determine the concentration of GSC to determine 8 mM as the final concentration of Met (**Supplementary Fig. 9**).


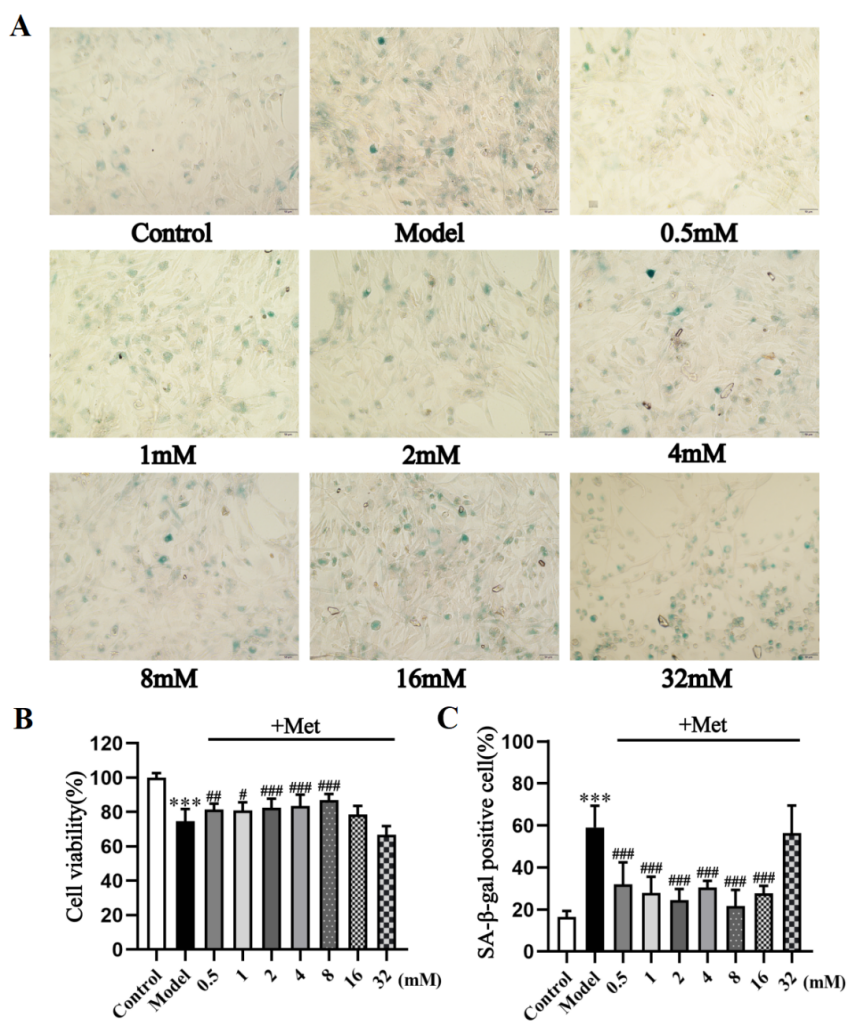


**Fig. 9 | Effect of different concentrations of Met on cell viability and cell senescence staining. A** The SA-β-gal staining of different concentrations of Met intervention for 24 hours (200x, scale at 50μm); **B** The cell viability rate of different concentrations of Met intervention for 24 hours; **C** Different concentrations of Met intervene in the staining ratio of cell senescence for 24 hours. Compared with the Control group, ****P*<0.001; Compared with the Model group, ^#^*P*<0.05, ^##^*P*<0.01, ^###^*P*<0.001.


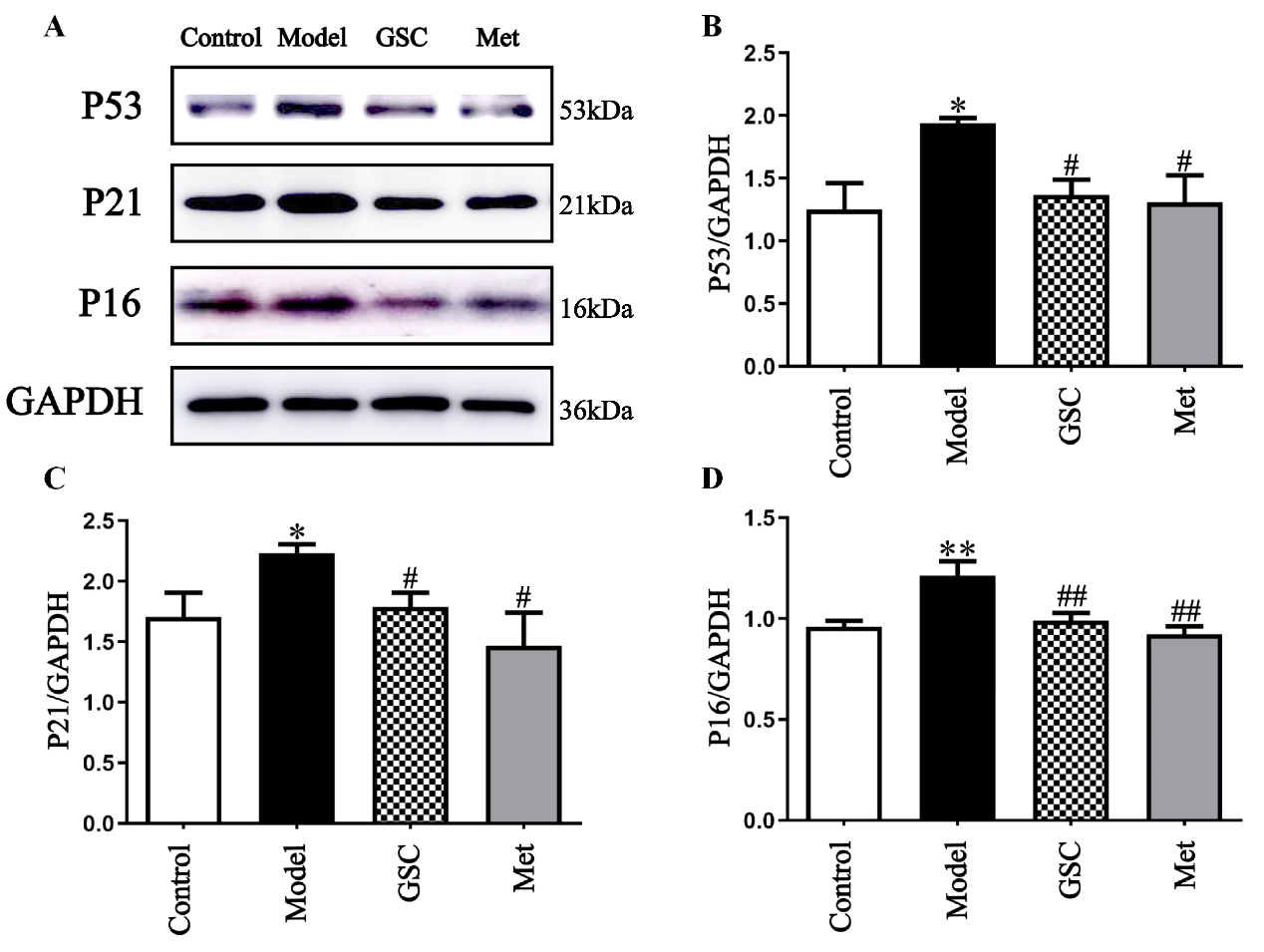


**Fig. 10 | Effect** **of GSC on the expression of senescence-related proteins in EPCs (n=3) A** WB bands showing the expression of P53, P21 and P16 proteins in each group; **B-D** Semiquantitative analysis of P53, P21, and P16 protein expression. Compared with that in the control group, **P* < 0.05, ***P* < 0.01; compared with that in the model group, ^#^*P* < 0.05, ^##^*P* < 0.01.

### Transfection and transplantation results of EPCs

72 hours after lentiviral transfection, the cells were placed under a fluorescence microscope, and a large number of green fluorescent EPCs were observed, as shown in **Fig. 11A**. The cell count and analysis were performed using ImageJ software, and the transfection rate was 71.01±8.8%. Under the detection of In Vivo Imaging System, the signal intensity changed with the lentivirus-labeled cells infected with different multiplicity of infection values (MOI), and the signal was stronger when the MOI value was 70, as shown in **Fig. 11B**. After the lentivirus-transfected EPCs were transplanted into mice through the tail vein, the light signal of the cells could be detected by the imaging system of small animals, indicating that the transplantation was successful (**Fig. 11C**).

**
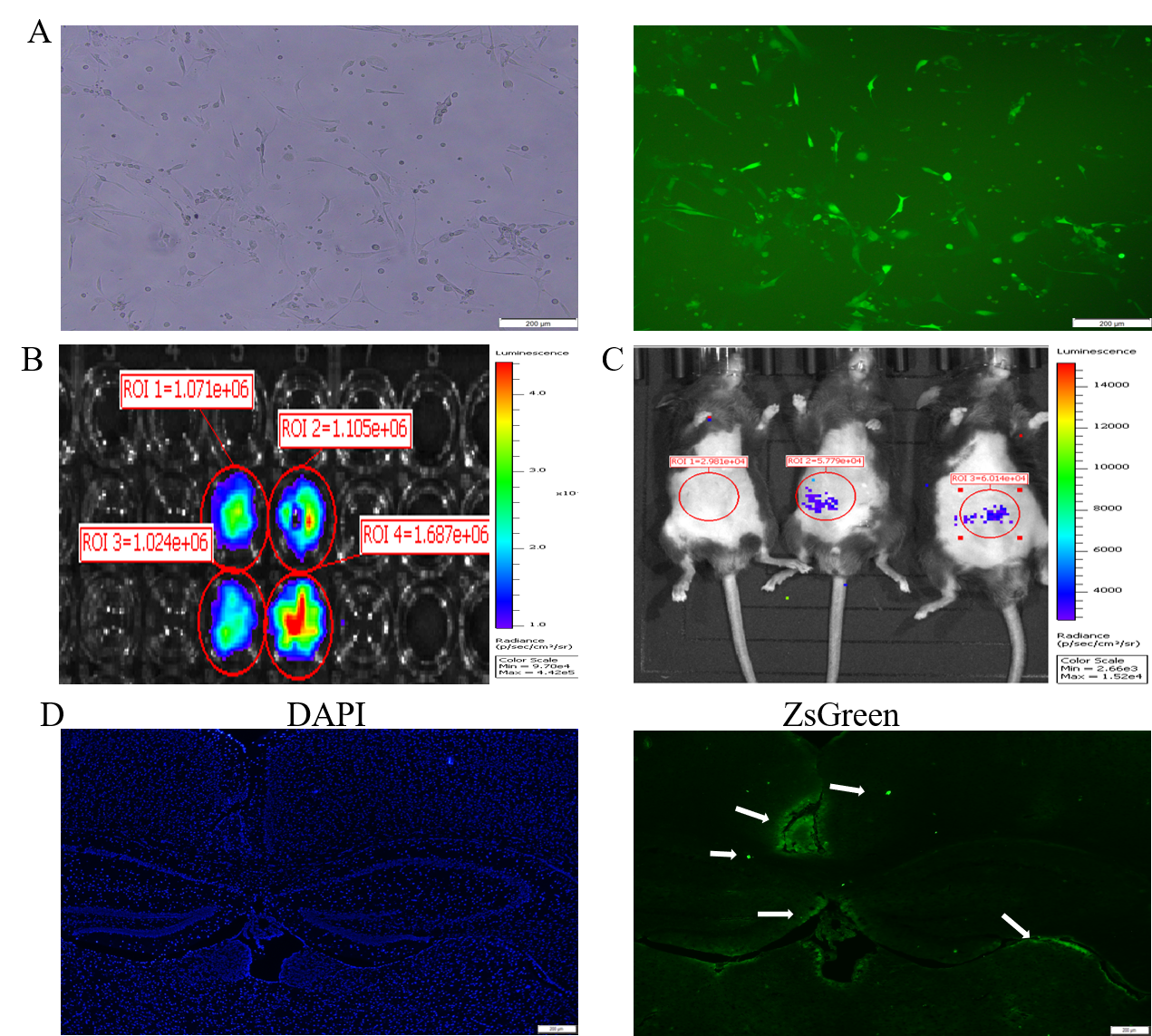
**

**Fig. 11 Transfection and transplantation results of EPCs. A**: Green fluorescent protein positive EPCs (100x); **B**: In vivo imaging of small animals to detect EPCs under different MOI values; **C**: Fluorescent imaging images of mice after EPCs transplantation under the small animal in vivo imaging system.
